# The Space Environment Activates Capsular Polysaccharide Production in Lacticaseibacillus rhamnosus Probio-M9 by Mutating the *wze* (*ywqD*) Gene

**DOI:** 10.1128/spectrum.04677-22

**Published:** 2023-03-02

**Authors:** Yue Sun, Shuai Guo, Jingfang Yang, Yingmeng Li, Zhihong Sun, Lai-Yu Kwok, Tiansong Sun, Wenjun Liu, Wenjun Liu

**Affiliations:** a Inner Mongolia Key Laboratory of Dairy Biotechnology and Engineering, Ministry of Agriculture and Rural Affairs, Inner Mongolia Agricultural University, Hohhot, Inner Mongolia, People’s Republic of China; b Key Laboratory of Dairy Products Processing, Ministry of Agriculture and Rural Affairs, Inner Mongolia Agricultural University, Hohhot, Inner Mongolia, People’s Republic of China; c Key Laboratory of Dairy Biotechnology and Engineering, Ministry of Education, Inner Mongolia Agricultural University, Hohhot, Inner Mongolia, People’s Republic of China; d Research and Development Department, Jiangzhong Pharmaceutical Co., Ltd., Nanchang, People’s Republic of China; Shenzhen Bay Laboratory

**Keywords:** space mutagenesis, *Lacticaseibacillus rhamnosus*, capsular polysaccharide, *wze*, *ywqD*, CPS gene cluster, *Lactobacillus*, gene cluster, genome analysis, mutagenesis

## Abstract

The study of microorganisms in outer space has focused mainly on investigating phenotypic changes in microbial pathogens induced by factors encountered in space. This study aimed to investigate the effect of space exposure on a probiotic bacterium, Lacticaseibacillus rhamnosus Probio-M9. Probio-M9 cells were exposed to space in a spaceflight. Interestingly, our results showed that a substantial proportion of space-exposed mutants (35/100) exhibited a ropy phenotype, characterized by their larger colony sizes and an acquired ability to produce capsular polysaccharide (CPS), compared with the original Probio-M9 or the ground control isolates without space exposure. Whole-genome sequencing analyses on both the Illumina and PacBio platforms revealed a skewed distribution of single nucleotide polymorphisms (12/89 [13.5%]) toward the CPS gene cluster, particularly in the *wze* (*ywqD*) gene. The *wze* gene encodes a putative tyrosine-protein kinase that regulates CPS expression through substrate phosphorylation. Transcriptomics analysis of two space-exposed ropy mutants revealed increased expression in the *wze* gene relative to a ground control isolate. Finally, we showed that the acquired ropy phenotype (CPS-producing ability) and space-induced genomic changes could be stably inherited. Our findings confirmed that the *wze* gene directly influences the capacity for CPS production in Probio-M9, and space mutagenesis is a potential strategy for inducing stable physiological changes in probiotics.

**IMPORTANCE** This work investigated the effect of space exposure on a probiotic bacterium, *Lacticaseibacillus rhamnosus* Probio-M9. Interestingly, the space-exposed bacteria became capable of producing capsular polysaccharide (CPS). Some probiotic-derived CPSs have nutraceutical potential and bioactive properties. They also enhance the survival of probiotics through the gastrointestinal transit and ultimately strengthen the probiotic effects. Space mutagenesis seems to be a promising strategy for inducing stable changes in probiotics, and the obtained high-CPS-yielding mutants are valuable resources for future applications.

## INTRODUCTION

Space microbiology, the study of microorganisms in outer space, is in its infancy worldwide, and microbial responses and adaptation to the space environment are interesting issues (1). The space environment has strong radiation, microgravity, and a hypervacuum, making it a more mutagenic environment than others with single mutagenesis conditions (2). Although the space environment is extreme and complex, microorganisms generally show high genetic and phenotypic adaptability (1). Thus, it is possible to generate microbes with novel and desired properties by exposing them to space, and space mutagenesis has gained much attention as a new cutting-edge technology in recent years. Previous space microbiology research has mostly focused on investigating pathogens, such as Staphylococcus aureus (3), Bacillus cereus (4), and Serratia marcescens (5), confirming that space mutagenesis is a feasible method for inducing various microbial physiological changes, e.g., biofilm formation ability, growth rate, and drug resistance (6). Such changes in pathogens may be detrimental to human health. On the other hand, if space mutagenesis is applied to beneficial microbes, it may hold promise for improving the probiotic properties of these microbes. For example, the species Ganoderma lucidum showed increased stress resistance and improved health-promoting effects after space mutagenesis (7). However, due to the fact that space mutagenesis is still not accessible to most researchers, there are only limited studies investigating the effects of space exposure on industrial and beneficial microbes.

Lactic acid bacteria (LAB) are widespread in nature and in diverse niches. Many LAB are known probiotics and are able to produce various types of health-promoting metabolites (8). For example, many probiotics can produce polysaccharides with nutraceutical potential and bioactive properties, which are in use in the food industry (9). Naturally, bacterial capsular polysaccharide (CPS) is produced as an important cell surface secondary metabolite. Not only does it enhance cell surface adhesion and biofilm formation to protect the cells from extreme conditions, but also, it can be converted into carbon or nitrogen sources to provide energy for supporting cell survival (10, 11).

Probiotics are live bacteria that confer a beneficial effect on the host when consumed in adequate quantities. Thus, the prerequisite of a successful probiotic is to be able to survive through the harsh environment encountered during the gastrointestinal transit. Producing CPS can improve the survival and colonization of probiotics in the host gut by enhancing their mucosal adhesion and competitiveness against the coexisting gut microbes. Probiotic-derived CPSs also offer health-promoting effects due to their antibacterial, immunomodulatory, and antitumor properties (12). A previous study found a 1.78-fold increase in exopolysaccharide (EPS) production in Lactobacillus confusus TISTR under high-salinity stress (13), indicating that a stressful environment could enhance EPS yield in this strain. It would therefore be of interest to see if an extremely stress-inducing environment like space could induce changes in CPS production. A few studies have successfully applied space mutagenesis to improve the health-promoting effects of probiotic bacteria. For example, a space-exposed mutant, Lactiplantibacillus plantarum SS18-5, has shown effective hypoglycemic effects on type 2 diabetes in a rat model (14). Another space-exposed mutant, Limosilactobacillus reuteri F-9-35, has been found to enhance anti-gastric injury and anticolitis effects in rats (15, 16). Therefore, space mutagenesis seems to be an interesting strategy for probiotic strain improvement. However, the space mutagenic effect has been found to vary between strains, genes, and chromosomal locations (17), and little is known about the effect of space exposure on the production of bacterial metabolites like CPS.

Lacticaseibacillus rhamnosus has become one of the most widely used LAB species due to its advantages over other species (18). *Lacticaseibacillus rhamnosus* Probio-M9 was isolated from a breast milk sample from a healthy woman and has good probiotic potential, exhibiting a high growth rate and strong tolerance to bile salts and artificial gastrointestinal fluids (19). Probio-M9 has shown effective antitumor effects in multiple rodent studies (20, 21) and a stress reduction effect in a cohort of graduate students (22). This study explored the effect of space exposure on Probio-M9, with the aim of inducing stable physiological changes in this bacterial strain. We characterized the phenotype, particularly induction of CPS production, of Probio-M9 space mutants by a combination of biochemical and multi-omics techniques.

## RESULTS

### Identification of single nucleotide polymorphisms (SNPs) in 117 isolates.

We prepared two parallel sets of cells of two growth conditions (streaking on de Man, Rogosa, and Sharpe [MRS] agar and submerging in 20% glycerol). One set of cells was used for the spaceflight mutagenesis experiment (samples were flown into space with a Long March 5B rocket), while the other one served as the ground control (kept at a simulated launch base without space exposure). After the spaceflight, single clones of space mutants and ground control isolates were picked for analysis.

Presumably, the space environment would induce substantial point mutagenesis, so we first compared the SNP profiles of the space mutants and ground control isolates. A total of 117 clones were picked for analysis (17 ground isolates from glycerol and 50 space mutants each from MRS agar and glycerol). The SNP profiles of 100 space mutant clones were determined by comparison against the reference Probio-M9 genome, identifying a total of 142 SNPs across all isolates located at 89 different sites. Twenty mutation sites lay within the intergenic regions, while 69 were distributed in the coding regions (corresponding to 18 synonymous, 47 nonsynonymous, and four nonsense mutations) (see Table S2 in the supplemental material). Far fewer SNPs (only seven chromosomally located) were identified across the 17 ground control isolates than in the space mutants. The average number of SNPs of the analyzed space mutants was 1.70 ± 1.15 (range, 0 to 14 SNPs per isolate). The HA-R7970-28 clone, a space mutant streaked on MRS agar, had the highest number of mutation sites.

**FIG 1:**
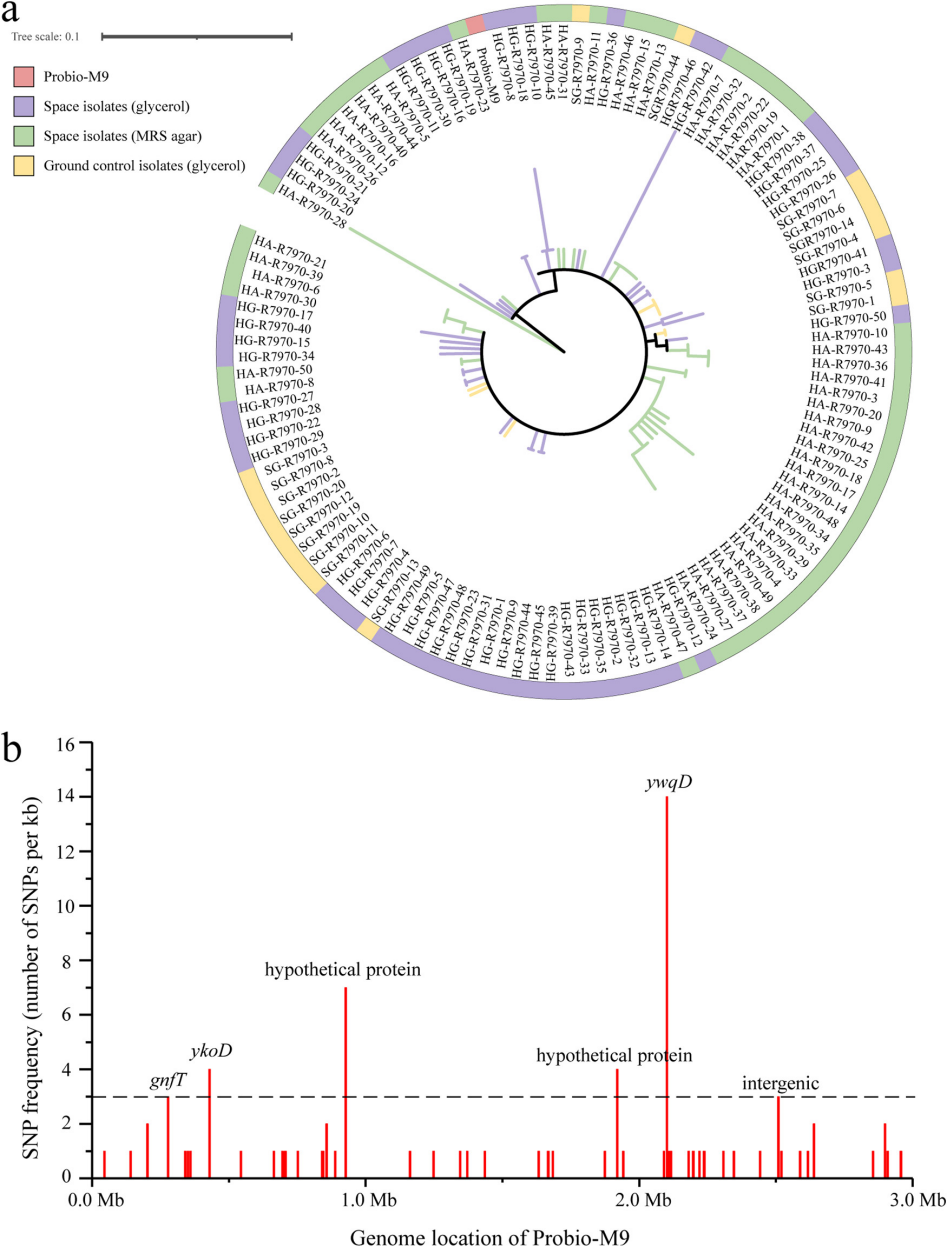
SNP analysis of 100 space mutant isolates and 17 ground control isolates with reference to the original Probio-M9 genome. (a) Phylogenetic tree of isolates constructed based on SNPs; (b) histogram showing the SNP distribution relative to the Probio-M9 genome. Six high-SNP-density regions (defined as genomic regions that had >3 SNPs per kb, indicated by the dashed line) were identified, including *gntT*, *ykoD*, *ywqD*, and two genes encoding hypothetical proteins, leading to mostly nonsynonymous mutations. One high-SNP-density region was in an intergenic region.

A phylogenetic tree was constructed based on the SNP distribution across isolates (Fig. 1a). A total of six high-SNP-density regions (defined as genomic regions that had >3 SNPs per kb) were identified, which comprised mostly nonsynonymous mutations. The skewed SNP distribution is suggestive of strong environmental selection pressure on the encoded function at these genomic regions (Fig. 1b). The *ywqD* gene was located in a high-SNP-density region that had the largest number of SNPs (35 SNPs), and only space isolates (35/142 SNPs, i.e., 24.6%), not any of the ground control isolates, were mutated in the *ywqD* gene. Four other genes (i.e., *gntT*, *ykoD*, and two genes encoding hypothetical proteins) were predicted at other high-SNP-density regions, and one of these regions was intergenic. The *gntT* gene is associated with high-affinity gluconate transport, involved in glucose-related metabolism. The *ykoD* gene encodes a thiamine import ATP-binding protein. The observation of a significantly higher mutation rate in the space mutants than in the ground control isolates confirmed that space exposure could induce point mutations in Probio-M9.

### Phenotype of space mutants.

**FIG 2:**
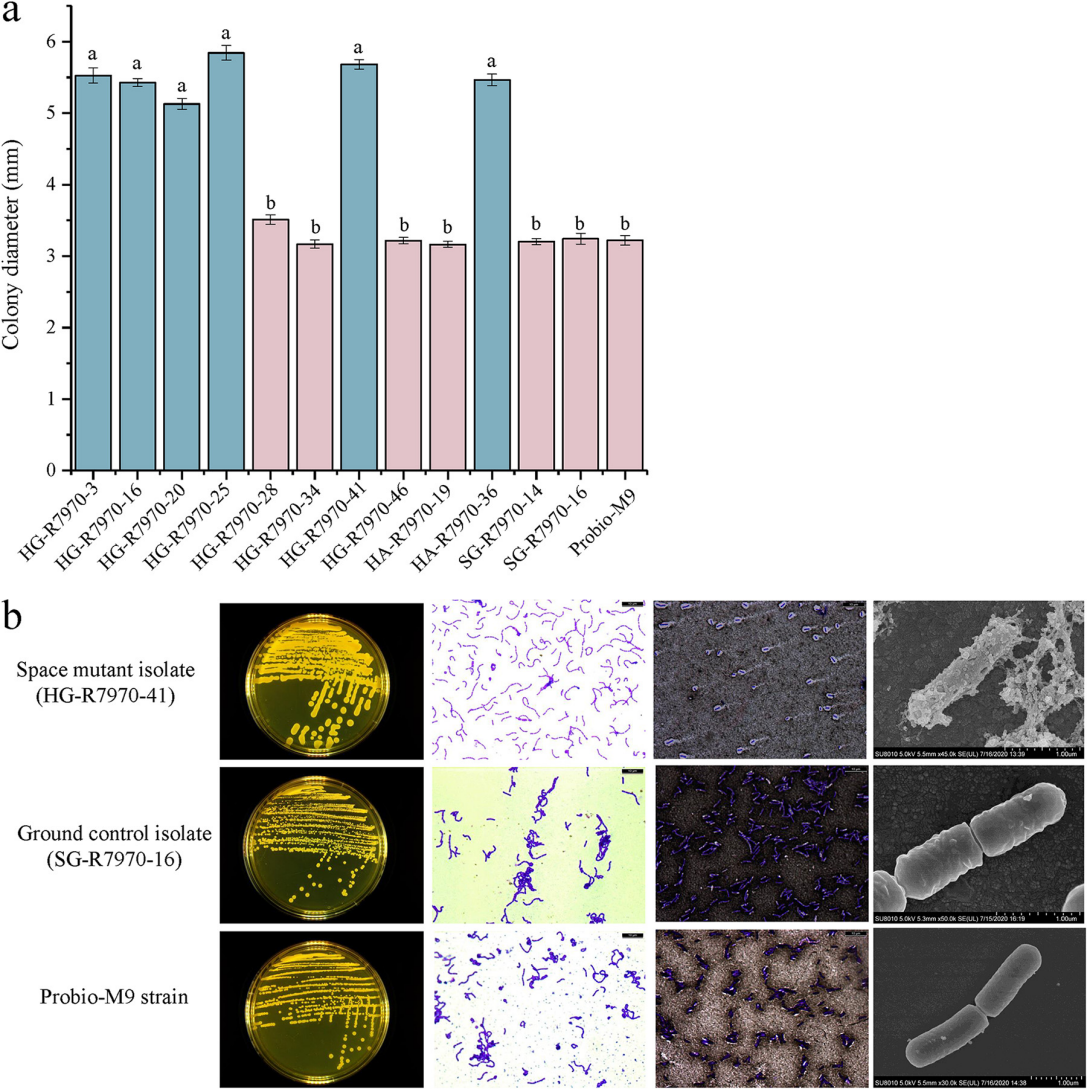
(a) Colony sizes of Probio-M9 and space and ground isolates grown on MRS agar for 48 h. Ropy and nonropy isolates are represented by blue and pink bars, respectively. Significant differences in colony diameters are indicated by different letters above the bars (*P < *0.05), and error bars represent standard deviations. (b) Morphology of Probio-M9 and representative space and ground isolates (HG-R7970-41 and SG-R7970-16, respectively) grown on MRS agar for 48 h, observed under light (Gram staining and India ink capsule staining; ×100 magnification) (left) and electron (×1,000 magnification) (right) microscopes.

A substantial proportion of space mutants had altered colony morphology, characterized by generally bigger sizes and colony ropiness. To further characterize them and to cover a range of phenotypically different space mutants, we selected 10 (of 100) clones of various sizes and colony morphologies for subsequent analysis. Six of them (HG-R7970-3, HG-R7970-16, HG-R7970-20, HG-R7970-25, HG-R7970-41, and HA-R7970-36) exhibited a ropy phenotype, while the other four formed nonropy colonies when grown on MRS agar. The colonies of the ropy clones were significantly larger than the colonies of the nonropy clones, the two ground control isolates, and Probio-M9 (*P < *0.05) (Fig. 2a). However, no significant difference was found between the colony diameters of the nonropy clones and those of the ground control isolates and Probio-M9 (*P > *0.05).

The microscopic morphology of a representative ropy space mutant (HG-R7970-41) was compared with that of a representative ground isolate (SG-R7970-16) and Probio-M9 (Fig. 2b). All space mutants and nonspace isolates were Gram stain positive. The nonropy space mutants (HG-R7970-28, HG-R7970-34, HG-R7970-46, HA-R7970-19) were morphologically similar to Probio-M9 and the ground control isolate (as short rods with regular ends that formed chains; no obvious capsule surrounding the cells after Indian ink polysaccharide staining). In contrast, the six ropy space mutants were strongly positive for cell capsules after Indian ink polysaccharide staining. Scanning electron microscopic analysis confirmed that the ropy mutants had more extracellular mucus and a rougher cell wall.

**FIG 3:**
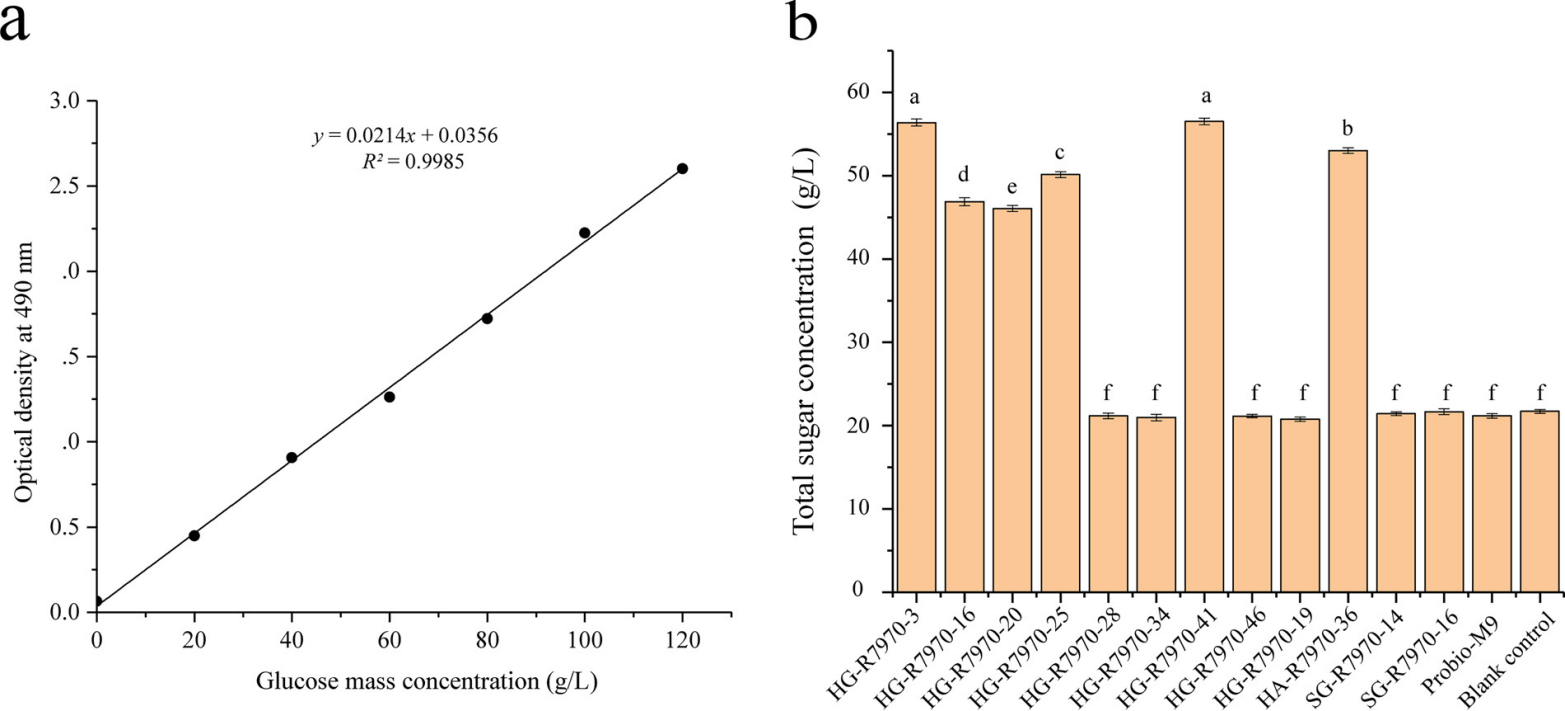
Glucose standard curve (a) and concentration of capsular polysaccharide (using glucose as a standard for measurement) of six ropy space mutants (b). Statistical differences in the concentration of capsular polysaccharide between mutants are represented by different letters above the bars (*P < *0.05). Error bars represent standard errors of the mean or standard deviations.

Next, we quantified the CPS (corresponding to the glucose concentration) produced by the 10 space mutants, the two ground control isolates, and Probio-M9. Exopolysaccharide production was observed only in the six ropy space mutants and not the other isolates or Probio-M9. Two of the ropy space mutants, HG-R7970-3 and HG-R7970-41, produced significantly more CPS than the other mutants (33.66 g/L and 33.78 g/L versus 23.36 g/L to 30.30 g/L; *P < *0.05) (Fig. 3).

### Whole-genome analysis by PacBio sequencing.

**FIG 4:**
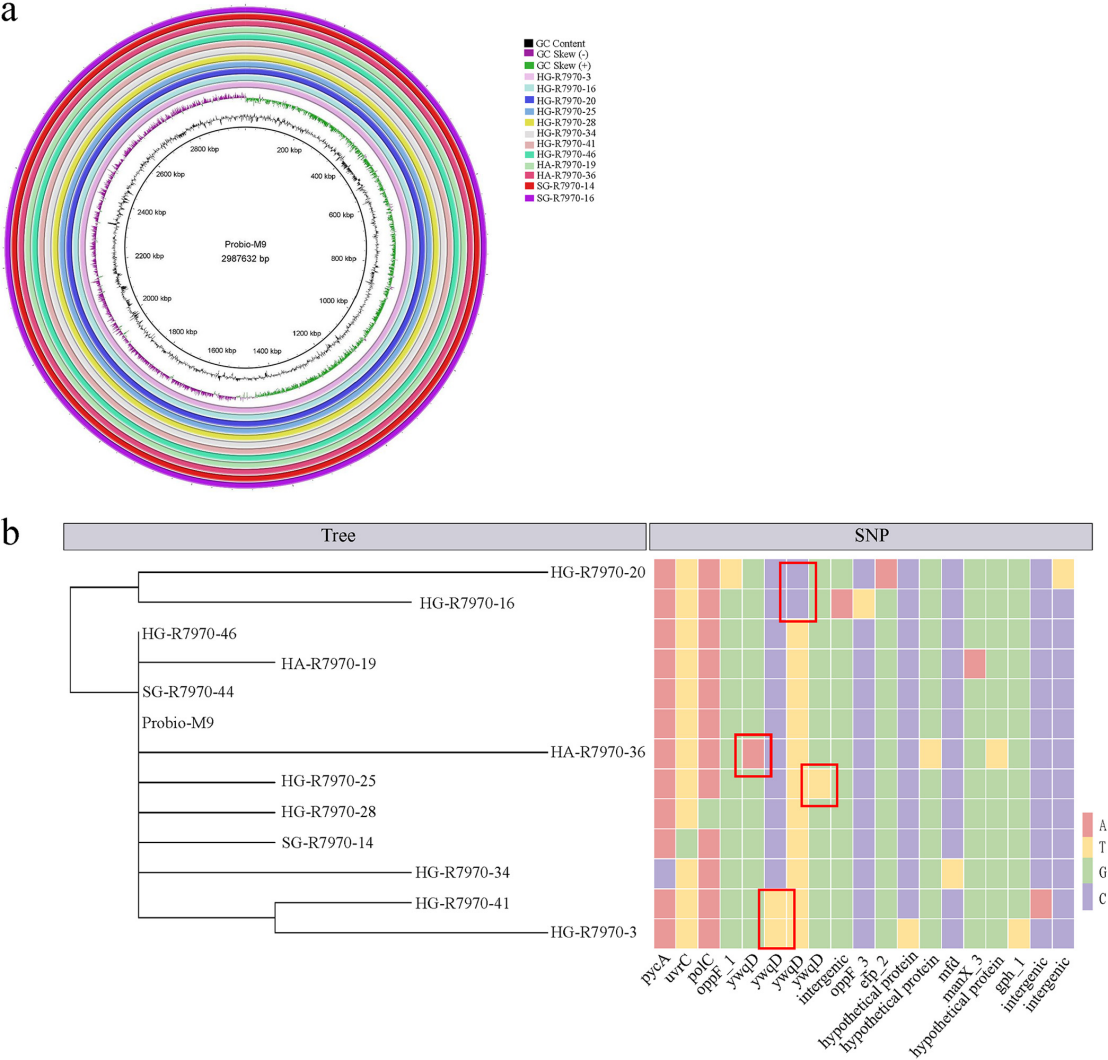
Genomic characteristics of space and ground isolates. (a) Genome maps of the space mutant isolates (HG-R7970-3, HG-R7970-16, HG-R7970-20, HG-R7970-25, HG-R7970-28, HG-R7970-34, HG-R7970-41, HG-R7970-46, HA-R7970-19, and HA-R7970-36) and the ground control isolates (SG-R7970-14 and SG-R7970-16) with reference to the original Probio-M9 genome. (b) Phylogenetic tree (left) and distribution of SNPs in various genes (right) in the isolates and Probio-M9. The four types of DNA nucleotides are represented by different colors. Red boxes highlight the SNPs in the *ywqD* gene.

The same 10 selected space mutants and two ground isolates were then subjected to PacBio whole-genome sequencing for precise mapping of their genomic changes. The PacBio-sequenced genomes were corrected with the Illumina genome data to improve the accuracy of genome mapping. Analysis with BRIG software revealed a high homology between Probio-M9 and the sequenced isolates (99.99%; no obvious difference in GC content and genome size) (Fig. 4a). A total of 19 SNPs were found across the 12 isolates (Table 1). Interestingly, all six ropy space mutants exhibited mutations at different positions of the *ywqD* gene, encoding the tyrosine-protein kinase in the CPS gene cluster (Fig. 4b), which is consistent with the results of SNP analysis by Illumina sequencing. Some space mutants (HG-R7970-3 and HG-R7970-41, D94N; HG-R7970-16 and HG-R7970-20, K70E) were found to share the same nonsynonymous mutations and were assigned to the same branch in the phylogenetic tree. The space mutants HA-R7970-36 (R97W) and HG-R7970-25 (S63Y) exhibited different SNPs in the *ywqD* gene. Interestingly, the mutants HG-R7970-3 and HG-R7970-41, which shared the same nonsynonymous mutations, produced significantly more CPS than mutants possessing SNPs at other locations in the *ywqD* gene.

**TABLE 1:** Distribution of SNPs in space mutants and ground control isolates^*a*^

Isolate	Nature of isolate (treatment, phenotype)	Mutation type	Gene	Gene description
HG-R7970-3	Space, ropy	Nonsynonymous	*ywqD*	Tyrosine-protein kinase
		Synonymous	—	Hypothetical protein
		Nonsynonymous	*gph1*	Phosphoglycolate phosphatase
HG-R7970-16	Space, ropy	Nonsynonymous	*ywqD*	Tyrosine-protein kinase
		Intergenic	—	
		Nonsynonymous	*oppF3*	Oligopeptide transport ATP-binding protein OppF
HG-R7970-20	Space, ropy	Nonsense	*oppF1*	Oligopeptide transport ATP-binding protein OppF
		Nonsynonymous	*ywqD*	Tyrosine-protein kinase
		Synonymous	*efp2*	Elongation factor P
HG-R7970-25	Space, ropy	Nonsynonymous	*ywqD*	Tyrosine-protein kinase
HG-R7970-28	Space, nonropy	Nonsynonymous	*polC*	DNA polymerase III PolC-type
HG-R7970-34	Space, nonropy	Nonsynonymous	*pycA*	Pyruvate carboxylase
HG-R7970-41	Space, ropy	Nonsynonymous	*ywqD*	Tyrosine-protein kinase
		Intergenic	—	
HA-R7970-19	Space, nonropy	Nonsynonymous	*manX3*	PTS system mannose-specific EIIAB component
HA-R7970-36	Space, ropy	Nonsynonymous	*ywqD*	Tyrosine-protein kinase
		Nonsynonymous	—	Hypothetical protein
		Nonsynonymous	—	Hypothetical protein
SG-R7970-14	Ground, nonropy	Nonsynonymous	*uvrC*	UvrABC system protein C

a —, the SNP lies within an intergenic region or the gene name is not available. No mutation site was detected in the space mutant HG-R7970-46 (nonropy) or the ground isolate SG-R7970-44.

As shown in Table 1, other nonsynonymous SNPs were distributed among various genes besides *ywqD*, including *gph1* and *oppF3* in the ropy space mutants, *pycA*, *polC*, and *manX3* in the nonropy space mutants, and *uvrC* in the ground isolate (SG-R7970-14). No mutation site was detected in the nonropy space mutant HG-R7970-46 and the ground isolate SG-R7970-16. Two nonsynonymous SNPs were also detected in two different genes encoding hypothetical proteins. Most of these nonsynonymous SNP-containing genes encode proteins of crucial physiological functions, including *pycA*, which encodes a DNA polymerase; *polC*, which encodes a pyruvate carboxylase, *manX3*, which encodes a phosphotransferase system mannose-specific EIIAB component, involved in carbohydrate transport and metabolism; *oppF3*, which encodes an oligopeptide transport ATP-binding protein, involved in cell membrane transport; and *gph1*, which encodes phosphoglycolate phosphatase, involved in lipid metabolism. However, these genes seem to be related more to the cell metabolism but not directly linked with the ropy phenotype.

Since all ropy mutants had a common feature, i.e., the acquisition of an SNP in *ywqD*, and provided that *ywqD* is involved in regulating the biosynthesis of CPS and that the amount of CPS production is specific to the SNP site in the *ywqD* gene, it is logical to postulate that the ropy phenotype and induction of CPS synthesis resulted from *ywqD* gene mutation.

### Identification of a complete CPS gene cluster in the Probio-M9 genome.

**FIG 5:**
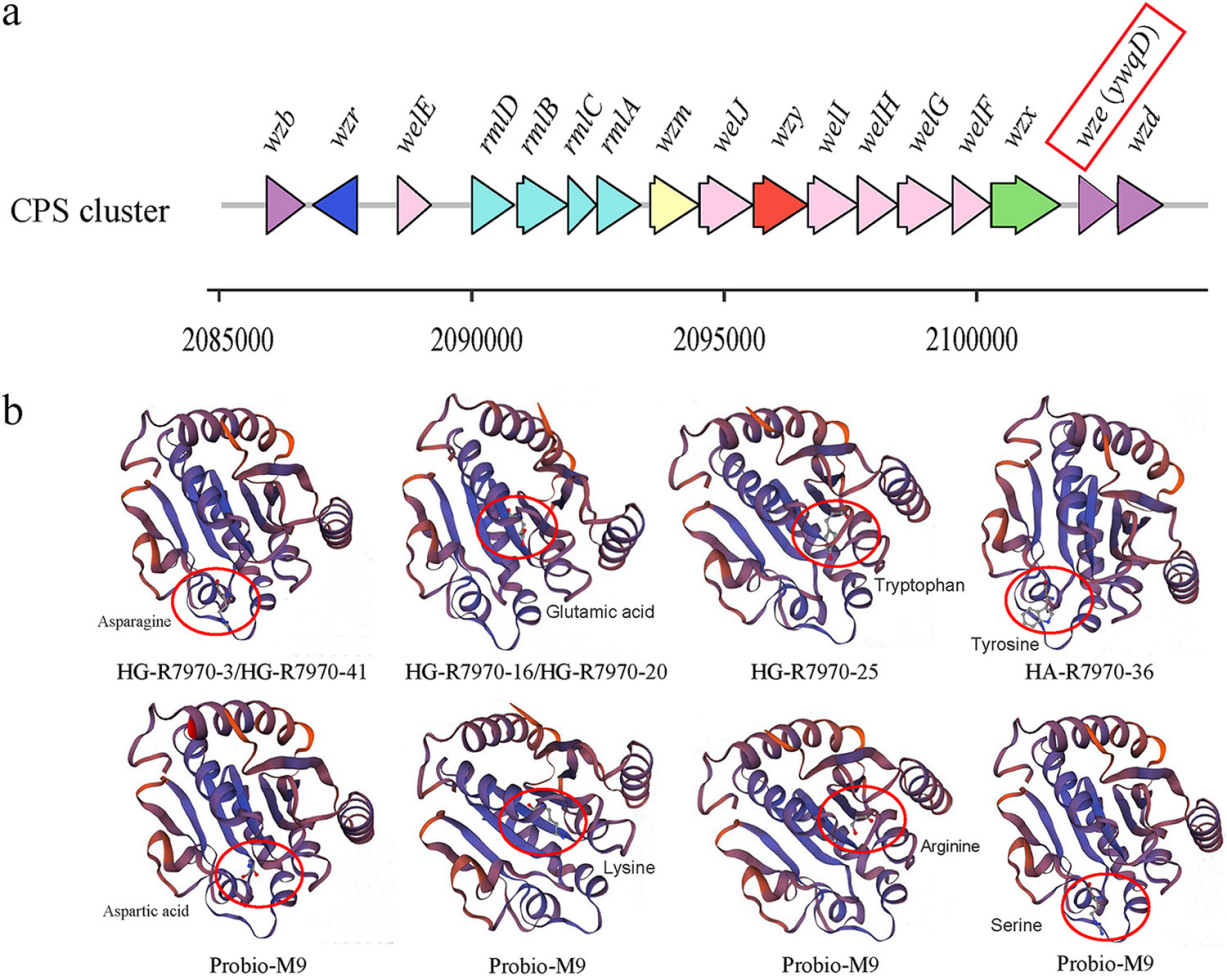
(a) Organization of the CPS gene cluster of Probio-M9. The putative function of the predicted gene products is represented by different colors: purple, proteins regulating CPS synthesis, particularly in chain length determination, encoded by *wzb*, *wzd*, and *wze*; blue, putative transcriptional regulator of polysaccharide biosynthesis, encoded by *wzr*; pink, glycosyltransferases for synthesizing polysaccharide repeat units, encoded by *welE*, *welF*, *welG*, *welH*, *welI*, and *welJ*; turquoise, enzymes constituting the dTDP-l-rhamnose biosynthesis pathway, encoded by *rmlA*, *rmlB*, *rmlC*, and *rmlD*; yellow, polysaccharide pyruvyl transferase, encoded by *wzm*; red, EpsG family polysaccharide polymerase, encoded by *wzy*; green, flippase, encoded by *wzx*. (b) Modeling of the tertiary structures of the Wze protein (a putative tyrosine protein kinase encoded by *wze*/*ywqD*) of the space mutants (top) in comparison with the Probio-M9 Wze protein (bottom) by SWISS-MODEL [https://swissmodel.expasy.org/interactive]. The red circle indicates the region of mutation in the predicted protein. Some isolates had the same mutation in the *wze* gene, including isolates HG-R7970-16 and HG-R7970-20 and isolates HG-R7970-3 and HG-R7970-41.

We then checked the sequences upstream and downstream of *ywqD* in the Probio-M9 genome and identified a complete CPS gene cluster, comprising 17 genes and spanning a genomic region of 18,018 bp (Fig. 5a). Multiple-sequence alignment of all sequenced mutants revealed no difference in the length and number of genes in the CPS gene cluster, and point mutations were identified exclusively within the *ywqD* gene. The predicted function, structural domains, and transmembrane helices of proteins encoded by each gene in the identified CPS cluster are shown in Table 2.

**TABLE 2:** Genes identified in the capsular polysaccharide cluster of *Lacticaseibacillus rhamnosus* Probio-M9

Seq_ID	Size (bp)	Designated gene	Predicted function	No. of transmembrane helices	Conserved domain within the protein	Accession no.
ORF2013	764	*wzb*	Tyrosine phosphatase	0	PHP superfamily	WP_024129339.1
ORF2014	893	*wzr*	Wzr/putative transcriptional regulator of polysaccharide biosynthesis	1	LytR_cpsA_psr superfamily	AAW22486.1
ORF2015	668	*welE*	Sugar transferase	1	Bac_transf	WP_015764590.1
ORF2016	842	*rmlD*	dTDP-4-dehydrorhamnose reductase	0	RmlD_sub_bind	WP_005712921.1
ORF2017	1025	*rmlB*	dTDP-glucose 4,6-dehydratase	0	RfbB	WP_005712923.1
ORF2018	572	*rmlC*	dTDP-4-dehydrorhamnose 3,5-epimerase	0	dTDP_sugar_isom	WP_015764591.1
ORF2019	872	*rmlA*	dTDP-glucose pyrophosphorylase	0	Glyco_tranf_GTA_type superfamily	AAW22479.1
ORF2020	989	*wzm*	Wzm/polysaccharide pyruvyl transferase	0	PS_pyruv_trans	AAW22478.1
ORF2021	1076	*welJ*	Glycosyltransferase family 4 protein	0	GT4_PimA-like	WP_015764593.1
ORF2022	1091	*wzy*	Wzy/EpsG family protein/putative polysaccharide polymerase	8	EpsG superfamily	WP_204148723.1
ORF2023	1019	*welI*	Glycosyltransferase family 2 protein	0	GT_2_like_d	WP_015764594.1
ORF2024	818	*welH*	Glycosyltransferase family 2 protein	0	GT2_RfbF_like	WP_015764595.1
ORF2025	1079	*welG*	Glycosyltransferase	0	Glycosyltransferase_GTB-type superfamily	WP_015764596.1
ORF2026	782	*welF*	Glycosyltransferase	0	GT2 superfamily	WP_015764597.1
ORF2027	1391	*wzx*	Oligosaccharide flippase family protein	14	MATE_like superfamily	WP_005712941.1
ORF2028	752	*wze* (*ywqD*)	CpsD/CapB family tyrosine-protein kinase/putative role in chain length determination	0	BY-kinase	WP_019728328.1
ORF2029	914	*wzd*	Wzz/FepE/Etk N-terminal domain-containing protein/chain length regulator	2	YveK superfamily	WP_015764599.1

Except for ORF2014 (corresponding to the *wzr* gene), all genes in the CPS cluster are in the same orientation (Fig. 5a). It is likely that the CPS gene cluster encodes the Wzx/Wzy-dependent pathway that produces heteropolysaccharide. The putative function of each gene was predicted based on its homologous functional domains and predicted transmembrane helices (Fig. 5a; Table 2). The three genes (*wzb*, *wzd*, and *wze*, corresponding to ORF2013, ORF2029, and ORF2028, respectively) encode proteins that regulate CPS synthesis, particularly in chain length determination. The *wze* gene (also known as *ywqD*) shows high homology to a CpsD/CapB family tyrosine-protein kinase. *wzr* (ORF2014) encodes a putative transcriptional regulator of polysaccharide biosynthesis. *welE*, *welF*, *welG*, *welH*, *welI*, and *welJ* (ORF2015, ORF2026, ORF2025, ORF2024, ORF2023, and ORF2021, respectively) encode glycosyltransferases (GTs) for synthesizing polysaccharide repeat units. *rmlA*, *rmlB*, *rmlC*, and *rmlD* (ORF2016 to ORF2019) encode enzymes that constitute a complete dTDP-l-rhamnose biosynthesis pathway. *wzm* (ORF2020) shows high homology (99%) to a polysaccharide pyruvyl transferase. *wzy* (ORF2022) encodes an EpsG-family polysaccharide polymerase. *wzx* (ORF2027) encodes a flippase, which has the highest number of transmembrane helixes among all predicted proteins. It might be important in transporting polysaccharide repeat units.

The YwqD protein comprises 250 amino acids, and the nonsynonymous mutations of YwqD of all six analyzed ropy space mutants lay in the N terminus of the protein (HG-R7970-3 and HG-R7970-41, D94N; HG-R7970-16 and HG-R7970-20, K70E; HG-R7970-25, S63Y; HA-R7970-36, R97W). A previous study found that replacing aspartate-81 and/or aspartate-83 (corresponding to aspartate-92 and -94 in the YwqD protein of Probio-M9) with alanines almost completely obliterated the ATPase activity of YwqD in Bacillus subtilis, and the purified mutant proteins (YwqD-81 and YwqD-83) did not exhibit any autophosphorylation activity (23). Interestingly, the two ropy space mutants with the highest CPS yield (HG-R7970-3 and HG-R7970-41) exhibited a D94N mutation, which is likely an active site required for proper protein functioning. The three-dimensional tertiary structures of the *wze/ywqD* gene products of Probio-M9 and six ropy space mutants were predicted and compared with the original YwqD protein of Probio-M9 (Fig. 5b), and, obviously, the mutant protein structures were altered by the nonsynonymous mutations. These results suggested that the activation of CPS production in Probio-M9 ropy space mutants is due to the modification of specific amino acids and/or the structure of the YwqD protein.

### Global and CPS gene expression in ropy space mutants at the transcriptomic and proteomic levels.

**FIG 6:**
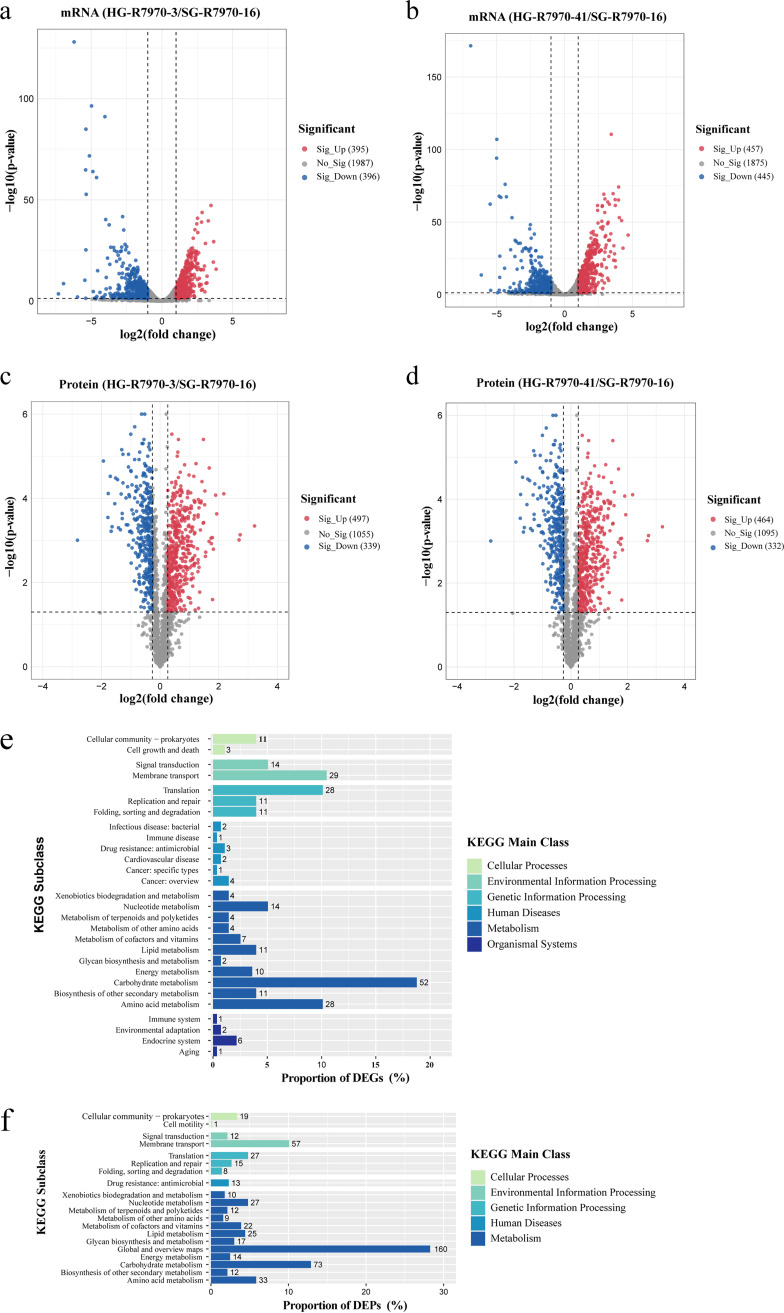
Volcano plots of the transcriptomics and proteomics data of the space mutants, HG-R7970-3 (a and c) and HG-R7970-41 (b and d), in comparison with the ground control isolate SG-R7970-16. The *x* axis indicates fold change in mRNA/protein expression (in log_2_ scale), and the *y* axis indicates the statistical significance of differential expression (*P* values were generated by *t* tests, shown in log_10_ scale). (e and f) Horizontal bar charts showing the distribution of differentially expressed genes (DEGs) and proteins (DEPs) across different Kyoto Encyclopedia of Genes and Genomes (KEGG) subclasses. The number next to each bar represents the number of DEGs and DEPs distributed to that KEGG subclass.

To gain further understanding of the molecular changes in the ropy mutants, particularly metabolic changes related to CPS production, the global and CPS gene expression of the two ropy space mutants with the highest CPS yield (HG-R7970-3 and HG-R7970-41) was comparatively analyzed with the ground isolate (SG-R7970-16) at the transcriptomic and proteomic levels (Fig. 6).

Dramatic changes were observed in the two space mutants at the transcriptomic and proteomic levels. At the transcriptomic level, HG-R7970-3 had 395 significantly upregulated and 396 significantly downregulated genes (Fig. 6a); HG-R7970-41 had 457 significantly upregulated and 445 significantly downregulated genes (Fig. 6b). At the proteomic level, HG-R7970-3 had 497 significantly upregulated and 339 significantly downregulated proteins (Fig. 6c); HG-R7970-41 had 464 significantly upregulated and 332 significantly downregulated proteins (Fig. 6d). The two space mutant isolates shared a large number of commonly up-/downregulated genes/proteins, including 347 upregulated and 329 downregulated genes and 421 upregulated and 299 downregulated proteins (Fig. S2). Some of these differentially expressed genes and proteins may be responsible for the production of CPS by the ropy space mutants. Annotation based on the Kyoto Encyclopedia of Genes and Genomes database revealed that most of these common differentially expressed genes/proteins belonged to the carbohydrate metabolism and membrane transport pathways (Fig. 6e and f), suggesting that the CPS production in these ropy space mutants is associated with alterations in carbohydrate metabolism.

Since the CPS gene cluster is responsible for bacterial CPS biosynthesis, we then focused on analyzing the differential expression of the CPS cluster genes and their encoded proteins. Obvious differential expression was observed in the CPS gene cluster in both ropy space mutants (Table 3). At the transcriptomic level, six CPS cluster genes of the space ropy mutants (*welE*, *rmlB*, *rmlC*, *rmlA*, *wzm*, and *wze*) showed increased expression, one gene (*wzy*) showed decreased expression, and 10 genes (*wzb*, *wzr*, *rmlD*, *welJ*, *welI*, *welH*, *welG*, *welF*, *wzx*, and *wzd*) exhibited no obvious variation, compared with the ground control isolate. At the proteomic level, two proteins (Wzb and Wzd) in the CPS cluster of the ropy space mutants showed increased expression, nine proteins (RmlD, RmlB, RmlA, WelJ, Wzy, WelI, WelH, WelG, and WelF) showed decreased expression, and six proteins (Wzr, WelE, RmlC, Wzm, Wzx, and Wze) exhibited no obvious variation, compared with the ground control isolate. Even though, generally, the transcriptional and translational expression of the CPS gene cluster in the two ropy space isolates showed a large discrepancy, the differential gene and protein expression patterns were highly consistent between the two mutants, confirming that similar physiological changes occurred in them after the space exposure and that such changes are likely associated with their common mutation in the *wze* gene.

**TABLE 3:** Differential expression of capsular polysaccharide cluster genes between space mutants and ground isolates^*a*^

Seq_ID	Putative protein	Fold change between isolates at level			
		HG-R7970-3 and SG-R7970-16		HG-R7970-41 and SG-R7970-16	
		Transcriptomic	Proteomic	Transcriptomic	Proteomic
ORF2013	Wzb	0.65	1.64	0.73	1.64
ORF2014	Wzr	0.53	1.07	0.54	1.05
ORF2015	WelE	2.06	0.95	2.20	0.98
ORF2016	RmlD	1.77	0.80	1.85	0.78
ORF2017	RmlB	2.43	0.82	2.23	0.80
ORF2018	RmlC	3.14	1.00	3.27	0.95
ORF2019	RmlA	3.23	0.68	3.14	0.66
ORF2020	Wzm	2.79	0.85	2.23	0.84
ORF2021	WelJ	0.99	0.58	0.87	0.54
ORF2022	Wzy	0.33	0.68	0.22	0.66
ORF2023	WelI	0.82	0.78	0.62	0.81
ORF2024	WelH	1.45	0.66	1.20	0.62
ORF2025	WelG	1.43	0.76	1.57	0.74
ORF2026	WelF	1.19	0.73	1.16	0.77
ORF2027	Wzx	0.91	0.99	1.03	0.91
ORF2028	Wze	2.07	0.83	2.36	0.86
ORF2029	Wzd	0.75	1.22	0.60	1.24

a HG-R7970-3 and HG-R7970-41 are space mutant isolates; SG-R7970-16 is a ground isolate.

### Genetic stability of space mutants.

**FIG 7:**
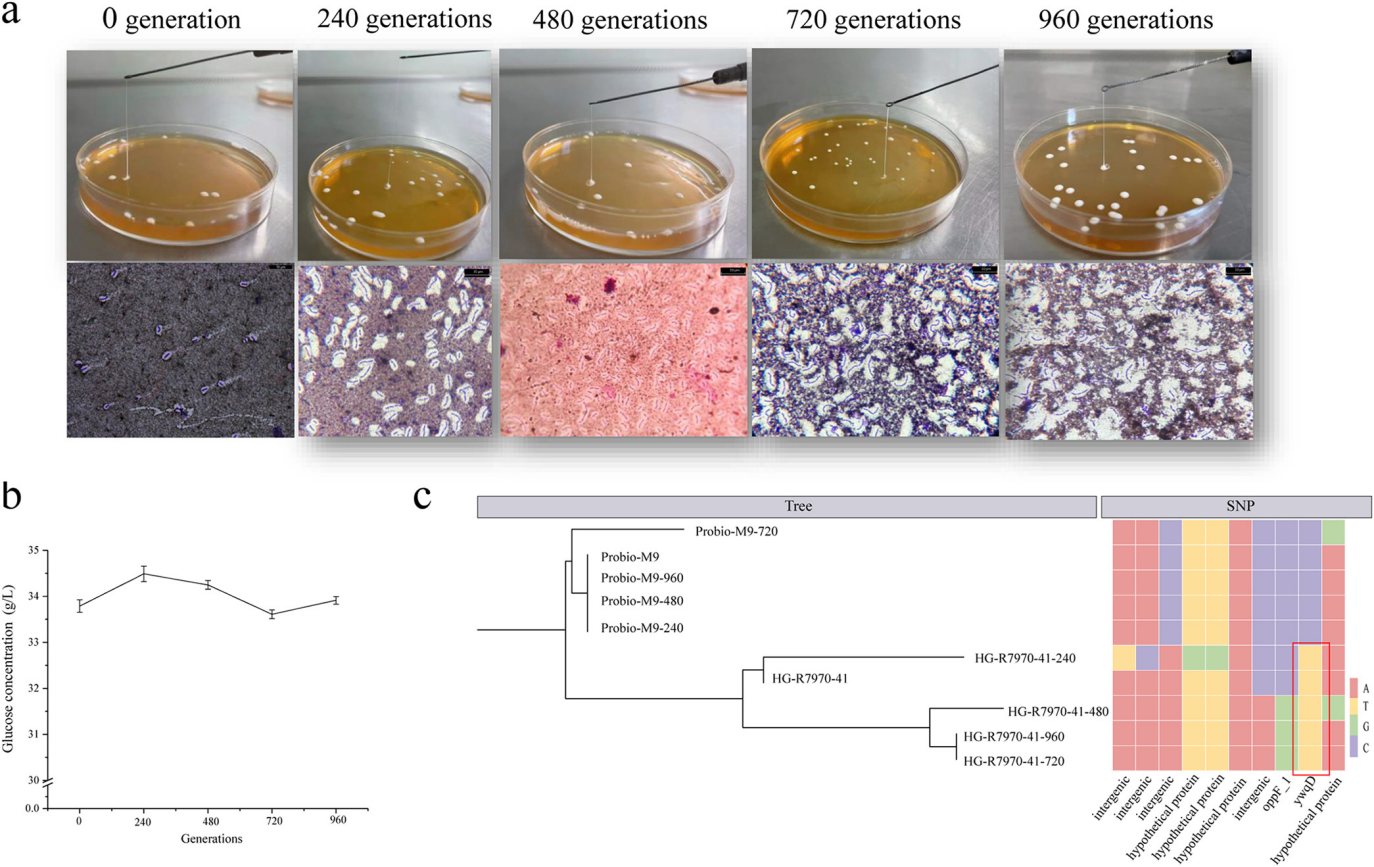
Phenotypic and genetic stability of HG-R7970-41. (a) The ropy phenotype of a selected space mutant (HG-R7970-41) and its corresponding microscopic morphology (India ink capsule staining) after continuous passage (illustrated by the generations of growth). (b) Capsular polysaccharide yield (using glucose as standard for measurement) after continuous passage. Error bars represent standard deviation (b). (c) Phylogenetic tree (left) and distribution of SNPs in various genes (right) in the mutant and Probio-M9 genomes after 0, 240, 480, 720, and 960 generations (c). The four types of DNA nucleotides are represented by different colors. The red box highlights the SNPs in the *ywqD* gene.

For realistic application, it would be necessary to ensure the phenotypic and genotypic stability of the space mutants. Thus, we evaluated the genetic stability of HG-R7970-41, the ropy mutant with the highest CPS yield. We inspected the ropy phenotype, capsule formation, CPS yield, and SNP stability in different generations during continuous passage. The ropy colony morphology, capsule formation, and CPS yield (>33 g/L throughout the passage) of the mutant HG-R7970-41 were stable (Fig. 7a and b). The SNP stability was inspected by resequencing cells of Probio-M9 and HG-R7970-41 after 0, 240, 480, 720, and 960 generations of subculture. Probio-M9 showed a higher stability, with only one new synonymous SNP detected after 720 generations of growth. In contrast, HG-R7970-41 gained more SNPs during passage. The two space-induced mutations in an intergenic region and within the *ywqD* gene were stably inherited throughout the cell passage (Fig. 7c). Therefore, the mutations induced by space exposure are genetically stable and are likely to cause long-term physiological changes to the mutants. Our data support the idea that space mutagenesis can be used to introduce novel characteristics, CPS production in this case, into Probio-M9.

## DISCUSSION

Space breeding by exposing plants to cosmic radiation and microgravity, generating crop varieties with diverse genotypes and phenotypes, has been developed as a strain improvement strategy. Space microbiology has focused mainly on studying the mutagenic effects of spaceflight on pathogens, and only very few works have investigated the impact of space exposure to beneficial microorganisms. Thus, this study carried out a space mutagenesis experiment on the probiotic strain *Lacticaseibacillus rhamnosus* Probio-M9 with the objective of finding out if this could be a feasible strategy for inducing stable physiological changes to probiotic bacteria, which may expand their potential applications in the future. We used integrated genomics, transcriptomics, and proteomics to characterize physiological changes in the obtained space mutants, particularly their phenotype and mechanism of CPS production.

Milojevic and Weckwerth summarized studies of microbial survival in and adaptation to the extreme space environment via global alterations in metabolic functions (24). Previous space mutagenesis experiments carried out in pathogens found that space exposure could result in different phenotypes. For example, one study showed that space exposure caused significant changes in bacterial drug resistance and gene expression in Escherichia coli (25). Another study found that a 33-day spaceflight decreased the biofilm formation ability of Acinetobacter baumannii (26). In contrast, a long-term spaceflight enhanced the biofilm formation ability and cell wall resistance to external environmental stress in Staphylococcus warneri while reducing sensitivity to chemical stimulation (6).

In our study, we analyzed 100 space mutants of Probio-M9. Interestingly, around one-third (35/100) of the space mutants showed a ropy phenotype with considerably larger colony size, which is related to their newly acquired ability to produce massive amounts of CPS. The fact that a relatively large proportion of Probio-M9 space mutants exhibited a similar acquired phenotype suggested that the space-induced mutations were not random. In particular, our SNP profiling revealed some high-SNP-density regions in the mutants, concentrated in the CPS gene cluster. A previous survival analysis of irradiated wild-type, acapsular mutant, and complemented mutant strains of Cryptococcus neoformans found that cryptococcal capsules contributed to radioprotection (27). The growth media (MRS agar and diluted glycerol) of Probio-M9 in our spaceflight experiment did not exert any specific environmental selection force. Thus, the mutagenesis was induced solely by the space environment. Considering the intense radiation field of galactic and solar origin and other extreme space factors like microgravity and hypervacuum, the directional mutation toward induction of CPS production could be a bacterial protective mechanism adopted against environmental extremes.

Apart from the CPS gene cluster, SNPs were mostly found in genes with functions related to cell transport, transcription, and metabolism. For example, OppF is a cellular transporter, and altering its gene could affect the proteolytic system in cells (28). ManX is an important component of the phosphotransferase system that participates in bacterial carbon metabolism (29). Some transcriptional regulatory genes were also mutated, which may be related to bacterial metabolism. The genotype-phenotype characteristics of other mutants remain to be further explored, and, based on previously reported observations and the results of this study, it seems that the effects of space mutagenesis vary greatly between bacterial species and strains.

Bacterial CPS production is an intrinsic protection mechanism against harsh external environments, such as desiccation and osmotic stress (29). Probio-M9 has been increasingly used in the industry for producing fermented milk and probiotics. In fermented milk production, a high EPS production of the starter and/or cofermentation bacteria not only improves the sensory quality and technological and rheological properties of yogurt but also enhance its functional properties, e.g., antioxidant and prebiotic potential (30). The CPSs produced by probiotic lactic acid bacteria are generally regarded as safe for applications in food and cosmetic industry (31), and many probiotic-derived CPSs have bioactive potential due to their antimicrobial, antitumor, antibiofilm, antiviral, anti-inflammatory, antidiabetes, immunomodulatory, and other desired activities (32). However, the CPS yield in LAB is generally low compared with that of other EPS-producing bacteria, and an EPS yield range of 10 to 15 g/L is recommended for feasible cost-effective production of EPS for use as a food ingredient (33). Our ropy space mutants (HG-R7970-3 and HG-R7970-41) did in fact gain a strong CPS-producing capacity, as they could synthesize up to 33.66 g/L and 33.78 g/L of CPS, respectively. Thus, acquisition of the capacity for CPS production in the naturally non-CPS-producing or weakly CPS-producing strain Probio-M9 is considered desirable, although the food technological properties and health-promoting effects of these ropy space mutants and their CPS still need to be further characterized.

The CPS gene cluster is highly conserved across different strains of *Lacticaseibacillus rhamnosus*, including LGG and ATCC 9595 (34, 35), suggesting that they have similar mechanisms of CPS synthesis. The Wzx-Wzy pathway is the main pathway for the synthesis of heteropolysaccharide in LAB (36). Similarly, in Probio-M9, a complete CPS gene cluster is present, which is likely the main pathway for CPS production in this strain. Our study found that mutations in the *wze* gene directly triggered the secretion of CPS, suggesting that the sites of *wze* gene mutations in the ropy mutants are potential regulatory sites that normally inhibit CPS production. The *wze* gene encodes a tyrosine kinase that promotes CPS synthesis through phosphorylation regulation (37). Further modeling of the tertiary protein structures of the original and the mutated Wze proteins predicted significant differences between them. A similar tyrosine phosphorylation-regulated mechanism of CPS production in Streptococcus pneumoniae has been reported. The tyrosine phosphorylation of the tyrosine-protein kinase of Streptococcus pneumoniae, CpsD, was found to correlate directly with the amounts of capsule produced. An increase in capsular material helps protect the cells from the host immunity, while a reduced level of CPS promotes a stronger binding to the host epithelial cells. The regulation of CPS production in Streptococcus pneumoniae helps the bacteria adapt to different niches in the host (38–40). Thus, the *wze* gene is likely responsible for regulating the CPS production in Probio-M9.

**FIG 8:**
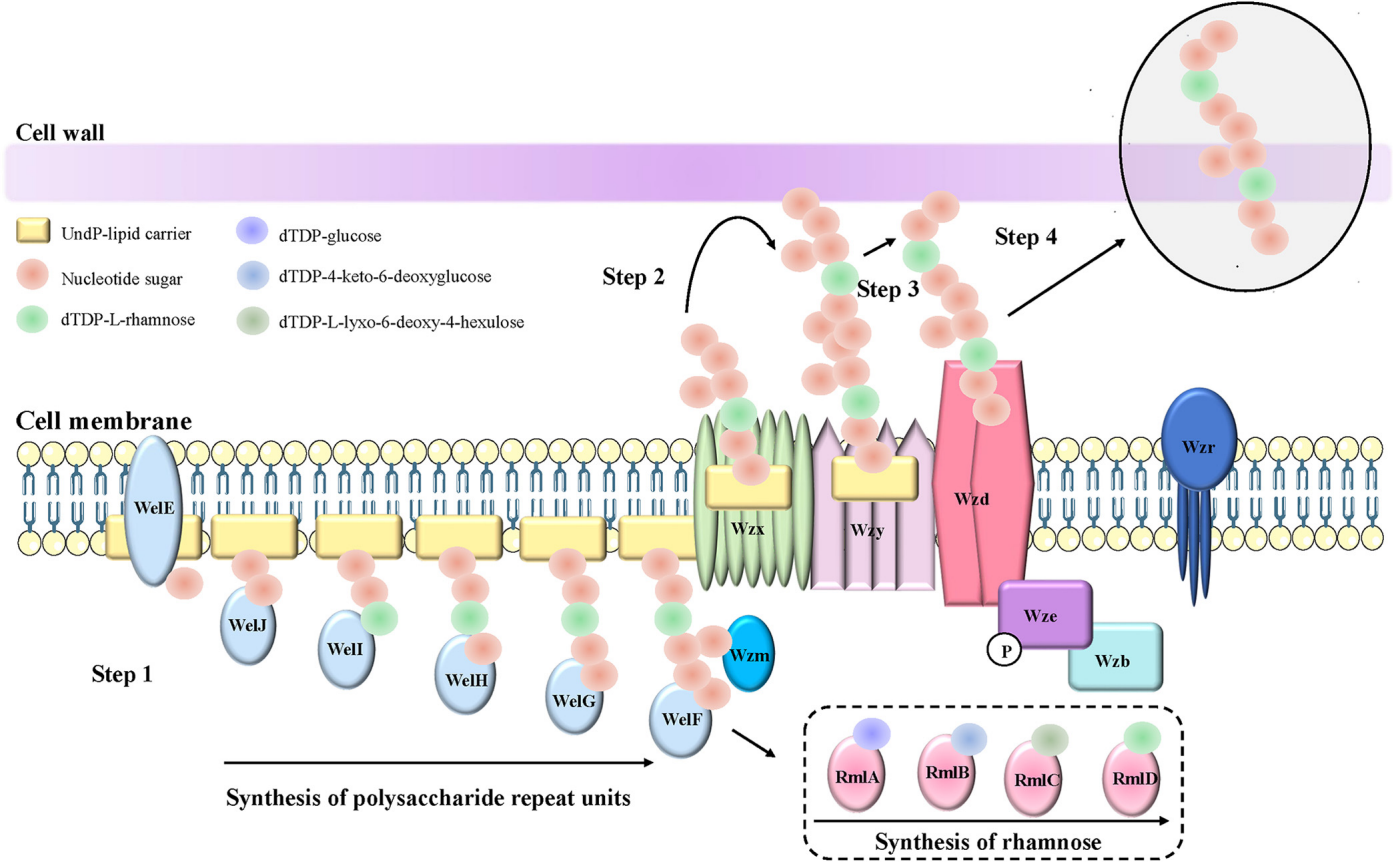
Schematic representation of the putative steps in CPS biosynthesis in *Lacticaseibacillus rhamnosus*. The main steps of CPS production are as follows. (Step 1) WelF, WelG, WelH, WelI, WelJ, and WelE (glycosyltransferases) add different nucleotide sugars to the undecaprenyl pyrophosphate (UndPP)-lipid carrier, which is located in the inner membrane. (Step 2) UndPP-linked polysaccharide repeat units are translocated from the inner to the outer leaflet of the inner membrane by the flippase, Wzx. (Step 3) Polysaccharide repeat units are polymerized by Wzy to form long polysaccharides. (Step 4) Wzd, Wzb, Wze, and Wzr regulate the polymerization and export of long polysaccharides. This figure is adapted from a previous study (39) with consideration of current transcriptomics and proteomics data.

Based on our transcriptomic/proteomic data and the existing CPS cluster literature, a proposed model of CPS biosynthesis was reconstructed (Fig. 8). Wzd-Wze-Wzb plays a regulatory role in polysaccharide synthesis as a protein complex (34, 41). Mutations in the *wze* gene in the ropy space mutants seem to have directly activated the Wze protein, which is consistent with the significant increase in *wze* at the mRNA level (fold change [FC] = 2.07 and 2.36 in the two ropy space mutants relative to the ground control). Moreover, in the process of polysaccharide synthesis, substrate phosphorylation by Wzy would indirectly change the structure and activity of the Wzd-Wze-Wzb protein complex, thereby affecting polysaccharide polymerization. The analyzed isolates were collected at the late logarithmic growth phase, when CPS production likely reached an equilibrium and protein feedback would inhibit the gene expression at the mRNA level (42). The inhibition of *wzy*, *wzd*, and *wze* expression at the mRNA level also supported our view. The *wzy* gene showed a significant decrease at the mRNA level (FC = 0.33 and 0.22 in the two ropy space mutants relative to the ground control), resulting in the inhibition of polysaccharide polymerization and affecting polysaccharide output.

Our results support the idea that the three regulatory proteins (namely, Wzd, Wze, and Wzb) mainly affect polymerization, enhancing the attachment of polysaccharide to the cell wall by regulating Wzy rather than the synthesis of polysaccharide repeat units. At the end of the logarithmic phase, the expression of genes associated with membrane-associated priming of the GT (WelE), dTDP-l-rhamnose synthesis, and polysaccharide pyruvyl transferase increased significantly at the mRNA level, indicating that polysaccharides were still being synthesized and that dTDP-l-rhamnose and pyruvyl were important components of CPS. However, the specific sequence of actions of the GTs needs further experimental verification. In short, the mutation in Wze possibly leads to corresponding changes in the protein complex (Wzd-Wze-Wzb), which further regulates the polysaccharide polymerization and yield. In addition, the mutation in Wze has no direct effect on the synthesis of polysaccharide repeat units and the function of the flippase (Wzx). Our results also prompted us to speculate that the mutants produced CPS mainly through *ywqD* gene feedback by regulating the expression of other genes in the CPS gene cluster.

Moreover, in the CPS synthesis process, the interactions between Wze, Wzb, and Wzd determine the export process of CPS, and the nonphosphorylated complex (Wzd-Wze-Wzb) may still allow polysaccharides to be released instead of attaching them to the cell wall (43), implying that isolates with a mutated *wze* gene could still synthesize polysaccharide repeat units. When Wze phosphorylates Wzd, interaction of Wzd with the polymerase may promote polysaccharide elongation (44). Therefore, the structure of polysaccharide before and after being exported to the cell wall is different. This variation is manifested in our study from the differential expression of gene encoding the GTs at the mRNA level. The GTs are mainly responsible for forming the polysaccharide repeat units. Moreover, the catch-and-release mechanism of Wzy polymerization is the result of interaction with Wzd (45). Thus, it is likely that the regulatory proteins can interact with each other to modulate the production of cellular polysaccharides, although the specific control mechanism is still unclear. Our results together suggest that the most probable mechanism of CPS induction is via the activation of Wze, through which the structure and function of Wzd and Wzb are affected. They are tripartite regulatory proteins controlling polysaccharide polymerization and export.

In this study, both transcriptomics and proteomics were used to analyze the differential expression of the CPS gene cluster of the ropy space mutants in comparison with the ground control isolates. We observed increased expression in most CPS cluster genes at the transcriptomic level, but a contrasting trend was seen at the proteomic level. Indeed, mRNA levels do not ultimately determine protein amounts, since translation of mRNAs can be influenced irrespective of mRNA levels (46). A previous study has also found a very low correlation between the transcriptomic and proteomic changes in Rhodobacter sphaeroides throughout its growth (47). Thus, the discrepancy between the transcriptomic and proteomic results seen in this study could simply be due to the fact that the amount of cellular protein and mRNA copy number of any gene may not always correlate with each other (48).

Our study demonstrated that space mutagenesis is a feasible approach for generating stable physiological changes in probiotic bacteria. In this study, we found that exposing Probio-M9 to space without imposing additional environmental selection forces induced an interesting and potentially useful ropy and CPS-producing phenotype in Probio-M9 due to *ywqD* gene mutation. It would be of interest in future studies to impose specific selection pressures in the growth media of the bacteria to attempt to direct the mutation toward desired traits for the purpose of probiotic strain improvement.

## MATERIALS AND METHODS

### Bacterial strain, cultivation conditions, and preflight sample preparation.

The strain Probio-M9 was originally isolated from breast milk of a healthy woman (19), and it was obtained from the Key Laboratory of Dairy Biotechnology and Engineering, Ministry of Education, Inner Mongolia Agricultural University. Bacteria were activated by inoculation and growth at 37°C for 24 h in MRS broth (Oxoid, Thermo Fisher Scientific, Inc., Basingstoke, UK). They were further subcultured three times in MRS medium at 37°C for 12 h. Activated cultures were stored at 4°C for use.

For preflight preparation, bacterial cells were washed in phosphate-buffered saline (8.0 g of NaCl/L, 0.2 g of KH_2_PO_4_/L, and 1.15 g of Na_2_HPO_4_/L; pH 7.2), before being streaked onto 1.5% MRS agar or transferred to 20% glycerol in water. Two replicate sets of Probio-M9 cultures were prepared for the space experiment and as the ground control. The detailed schedule for the spaceflight experiment is shown in Table S1. Briefly, the set of cells for the space experiment was flown into space with a Long March 5B rocket under the following flight conditions: orbital inclination of 41.01, initial orbital altitude of 324.2 km, orbit elevation to 7,970 km after several orbital changes, orbital flight for 2 days 19 h with consistent cabin air composition with atmosphere, total pressure range of 81 kPa to 105 kPa, and return cabin radiation dose of 128 rad. The ground control cells were kept at a simulated launch base, where the external conditions were identical to those of the space experiment cultures except that they were not exposed to the space environment. After the end of the space flight, all four cultures were transported to the laboratory at 4°C.

### Isolation of single colonies.

Bacteria on the two MRS agar plates (from space experiment and ground control) were gently scraped and resuspended in a small amount of normal saline. Together with the two glycerol bacterial suspensions, 0.5 mL of each of these four samples was serially diluted to 10^−5^, 10^−6^, and 10^−7^ in 4.5 mL of phosphate-buffered saline, and 200 μL of each sample was plated on triplicate sets of modified MRS agar and incubated aerobically at 37°C for 48 h. Fifty single colonies were selected from each of the space experiment cultures (designated HA-R7970-1 to HA-R7970-50 and HG-R7970-1 to HG-R7970-50 for clones recovered from the cultures on MRS agar and in glycerol, respectively), and 17 single colonies were selected from the ground control culture in glycerol (designated SG-R7970-1 to SG-R7970-17) for genome resequencing.

### Screening and morphological characteristics of space mutants.

To cover mutants of potentially different phenotypes and characteristics (Fig. S1), we selected independent colonies grown on MRS agar in various sizes and morphologies for subsequent PacBio single-molecule real-time (SMRT) analysis together with the original Probio-M9 strain. A total of 10 space mutants (HA-R7970-19, HA-R7970-36, HG-R7970-3, HG-R7970-16, HG-R7970-20, HG-R7970-25, HG-R7970-28, HG-R7970-34, HG-R7970-41, and HG-R7970-46) and two ground control isolates (SG-R7970-14 and SG-R7970-16) were picked. The Probio-M9 strain and the 12 selected clones were subcultured twice on MRS agar plate by incubating anaerobically for 48 h. A colony analyzer was used to measure the single-colony diameters at 24 h and 48 h (reported as means and standard deviations [SD]). Single colonies were picked with an inoculation loop to observe whether they exhibited a ropy phenotype and for Gram staining. The cell morphology was observed under a microscope and by scanning electron microscopy (49). India ink capsule staining was used to observe the cell capsule, and a positive result for capsule formation was characterized by a clear and refractile area surrounding the cell against a dark background (50).

### Genomics.

**(i) Whole-genome Illumina sequencing.** To identify genome-level variation between bacterial clones, whole-genome Illumina sequencing was performed. Genomic DNA was extracted from 100 selected space mutants and 20 ground control isolates by the sodium dodecyl sulfate method (51). Sequencing libraries were generated using the NEBNext Ultra DNA library preparation kit for Illumina (New England Biolabs, Inc., Ipswich, MA, USA) following the manufacturer’s recommendations. The extracted DNA samples were fragmented by sonication to a size of around 350 bp, and the DNA fragments were end polished, A tailed, and ligated with the full-length adaptor for Illumina sequencing. The whole genomes of all isolates were sequenced on an Illumina NovaSeq PE150 system at the Beijing Novogene Bioinformatics Technology Co., Ltd. (Beijing, China). The average coverage depth of high-quality data was over 300-fold for each sample. All high-quality paired reads were assembled using the SOAPdenovo2 assembler (52).

**(ii) Whole-genome PacBio SMRT sequencing.** The PacBio SMRT sequencing technology was further used to sequence the genomes of 10 selected space mutants and two ground control isolates. The DNA of each space mutant or ground isolate was extracted using the Promega DNA extraction kit (Pacific Biosciences Inc., Menlo Park, CA, USA) using the standard protocol. DNA libraries were constructed with the SMRTbell template preparation kit (Pacific Biosciences Inc., Menlo Park, CA, USA). Completed libraries were then bound to proprietary P6 v2 polymerase and sequenced using C4 chemistry (on a PacBio RS II instrument) in the circular consensus sequencing mode in one flow cell. The sequence reads underwent *de novo* assembly using the R PacBio hierarchical genome assembly process (RS_ HGAP_Assembly.3). Circlator was used to cyclize the assembled data (53). The PacBio-SMRT sequencing data were corrected using the assembly results of Illumina sequencing to achieve the complete genome data set (54).

**(iii) Genome analysis.** Contigs of each isolate were aligned to the original Probio-M9 genome to identify SNPs using MUMmer 3.0 (55). The criteria of SNP screening were as follows: the average base calling error rate was <0.01; sites were covered by >10 paired-end reads; and identified sites did not fall within the repetitive regions of the genome. Detailed genomic maps of the 12 sequenced isolates were compared against the original Probio-M9 genome using BRIG software (56). The genomes of all isolates and the original Probio-M9 were functionally annotated with the Prokka software using default parameters (57). Manual BLASTp searches were used to find enzyme/protein homologues (query coverage > 95%; identity > 95%). Searches for conserved domains within a protein or coding nucleotide sequence were carried out using the Conserved Domains Database at the National Center for Biotechnology Information (http://www.ncbi.nlm.nih.gov/cdd). Transmembrane domains were predicted with the TMHMM server v. 2.0 (http://www.cbs.dtu.dk/services/TMHMM/). SWISS-MODEL was used to predict protein tertiary structure (https://swissmodel.expasy.org/interactive). Genes of CPS clusters were annotated according to the methods described in a previous study (35). These genes were named based on the bacterial polysaccharide gene nomenclature system (58). Multiple-sequence alignment was performed using Clustal-W program online analysis (http://www.ebi.ac.uk/Tools/clustalw/). Based on the results for SNPs, FastTree software (59) was used to build an adjacent tree (neighbor-joining tree), which was visualized by iTOL (Interactive Tree of Life; https://itol.embl.de/).

**(iv) Capsular polysaccharide content.** The CPS content of the 12 isolates and Probio-M9 were measured in triplicate by inoculating (2% [vol/vol], i.e., approximately 10^6^ CFU/mL) and cultivating the cells in 10 mL of MRS medium at 37°C for 12 h. Then, cell culture supernatant of each culture was separately collected by centrifugation at 4°C for 20 min (4,000 rpm). Each supernatant sample was mixed with 80% (vol/vol) trichloroacetic acid to achieve a final sample concentration of 4%. The mixture was allowed to stand at 4°C for 10 h before being centrifuged at 4,000 rpm for 20 min. The supernatant was mixed with 3 volumes of absolute ethanol, let stand at 4°C for 24 h, and then centrifuged (4,000 rpm, 20 min). The precipitate was reconstituted with a small amount of distilled water and dialyzed in a dialysis bag with an 8,000- to 14,000-Da pore size for 48 h against water (change of water every 4 h). The quantity of CPS was determined by measuring the total sugar content in the precipitates by the phenol-sulfuric acid method (60) using glucose as a standard. Uninoculated MRS broth was processed in parallel as the blank control. The CPS content (reported in grams per liter) was the difference between total sugar content of the cell culture supernatant and that of the blank control.

### Transcriptomics.

**(i) RNA extraction and sequencing.** Transcriptomic analysis was performed on two space mutants (HG-R7970-3 and HG-R7970-41) with the highest EPS yield and one ground control isolate (SG-R7970-16). The chosen isolates were cultured to the end of logarithmic phase (12 h) for RNA extraction by using the TRIzol reagent (Invitrogen, California, USA), and sample preparation was performed in triplicate. A transcriptome sequencing (RNA-seq) transcriptome library was prepared with the Illumina TruSeq RNA sample preparation kit (Illumina, San Diego, CA, USA). The paired-end RNA-seq sequencing library was sequenced with the Illumina HiSeq X 10 (2 × 150-bp read length). Raw sequences were processed using the Illumina GA pipeline (version 1.6) to obtain paired-end reads of 150 bp.

**(ii) Transcriptome analyses.** First, low-quality bases were filtered to obtain clean data. The clean reads were then compared with the reference genome sequence of Probio-M9 using Bowtie 2 (61). Differentially expressed genes were identified by RSEM (http://deweylab.biostat.wisc.edu/rsem/), Kallisto (https://pachterlab.github.io/kallisto/), and Salmon (https://combine-lab.github.io/salmon/). Differential gene expression was calculated by the EdgeR and DESeq2 packages in R (version 4.2.1) (62). Genes with *P* values of <0.05 and FCs of >2 were considered significantly upregulated, while those with *P* values of <0.05 and FCs of <0.5 were considered significantly downregulated.

### Proteomics.

**(i) Total protein extraction and tandem mass tag labeling.** Proteome analysis was performed on the two space mutants (HG-R7970-3 and HG-R7970-41) that had the highest EPS production and one ground control isolate (SG-R7970-16). These isolates were cultured to the end of logarithmic phase (12 h) for protein extraction. All proteins in the samples were extracted and labeled using the methods described in a previous study (63), and the sample preparation was performed in triplicate. Briefly, bacterial cells were collected by centrifugation, and then the cell pellets were frozen in liquid nitrogen before addition of the lysis buffer and subjected to cold methanol protein precipitation. A bicinchoninic acid (BCA) protein assay kit (Beyotime Biotechnology, Inc., Nantong, China) was used for protein quantification. Trypsin was added at a 1:50 trypsin-to-protein mass ratio and incubated at 37°C overnight. Trypsin-digested peptides were labeled with a 10-plex tandem mass tag reagent (catalog no. 90111; Thermo Fisher Scientific, Inc., Waltham, MA, USA) according to the manufacturer’s instructions.

**(ii) LC-MS/MS.** Pooled protein samples from each isolate were fractionated by Acquity ultraperformance liquid chromatography (UPLC; Waters, Milford, MA, USA) with Acquity UPLC BEH C_18_ columns (1.7 μm, 2 by 150 mm; Waters, Milford, MA, USA) to increase proteome depth. Labeled peptides were analyzed by online nanoflow liquid chromatography-tandem mass spectrophotometry (LC-MS/MS) performed on an EASY-nLC system (Thermo Fisher Scientific, Inc., Waltham, MA, USA) connected to a Q Exactive quadrupole Orbitrap mass spectrometer (Thermo Fisher Scientific, Inc., Waltham, MA, USA) through a nanoelectrospray ion source. Briefly, the C_18_ reversed-phase column (75 μm by 25 cm; Waters, Milford, MA, USA) was equilibrated with solvent A (2% formic acid in 0.1% formic acid) and solvent B (80% acetonitrile in 0.1% formic acid). Peptides were eluted using the following gradient at a flow rate of 300 nL/min: 0 to 1 min, 0% to 5% B; 1 to 63 min, 5% to 23% B; 63 to 88 min, 23% to 48% B; 88 to 89 min, 48% to 100% B; and 89 to 95 min, 100% B. The Q Exactive instrument was operated in the data-dependent mode to automatically switch between full-scan MS and MS/MS acquisition. Full-scan MS spectra (350 to 1,300 *m/z*) were acquired in the Orbitrap mass analyzer with a resolution of 70,000 after ion accumulation to the target value (10^6^) based on predictive automatic gain control of the previous full scan. Dynamic exclusion was set to 18 s. The 20 most intense multiply charged ions (*z* ≥ 2) were sequentially isolated and fragmented in the octopole collision cell by higher-energy collisional dissociation at a resolution of 35,000 for the fast-scanning method (63).

**(iii) Protein identification.** ProteomeDiscover software version 2.1 (Thermo Fisher Scientific, Inc., Waltham, MA, USA) was used to analyze the raw data. A target false discovery rate threshold of 1% was set at the high-confidence peptide level, so that only proteins with at least one unique peptide were identified. Differentially expressed proteins were defined by an FC of >1.2 or <0.83 and a *P* value of <0.05.

### Genetic stability.

Bacterial cells of each isolate and Probio-M9 were subcultured twice in MRS broth at 37°C for 12 h, followed by continuous propagation (1% [vol/vol]) in fresh MRS medium every 24 h. A 100-fold daily increase in bacterial growth was roughly equal to 6.6 generations for each subcultivation (64). Bacterial cells were collected at 0, 240, 480, 720, and 960 generations for genome sequencing and phenotypic observation for changes in cell morphology, capsular membrane, and CPS production.

### Statistical analysis.

All statistical analysis and plots were generated by R (version 4.2.1) unless otherwise stated. Significant differences between isolates were evaluated by Wilcoxon and Kruskal-Wallis tests using the R package ggpubr (https://github.com/kassambara/ggpubr/) unless otherwise stated. Phylogenetic trees and heat maps of SNPs were constructed using the ggtree and ggmsa packages in R software, respectively (65). The CPS gene cluster was plotted by the gggenes R package. The volcano plots were drawn using OmicStudio (https://www.omicstudio.cn/tool).

### Data availability.

The whole-genome sequencing and RNA-seq data generated by this study have been deposited in the National Center for Biotechnology Information database under the BioProject accession number PRJNA889467.

## References

[1] Liu C. 2017. The theory and application of space microbiology: China's experiences in space experiments and beyond. Environ Microbiol 19:426–433. doi:10.1111/1462-2920.13472. 27459305

[2] Horneck G, Klaus DM, Mancinelli RL. 2010. Space microbiology. Microbiol Mol Biol Rev 74:121–156. doi:10.1128/MMBR.00016-09. 20197502 PMC2832349

[3] Guo J, Han N, Zhang Y, Wang H, Zhang X, Su L, Liu C, Li J, Chen C, Liu C. 2015. Use of genome sequencing to assess nucleotide structure variation of Staphylococcus aureus strains cultured in spaceflight on Shenzhou-X, under simulated microgravity and on the ground. Microbiol Res 170:61–68. doi:10.1016/j.micres.2014.09.001. 25304992

[4] Su L, Zhou L, Liu J, Cen Z, Wu C, Wang T, Zhou T, Chang D, Guo Y, Fang X, Wang J, Li T, Yin S, Dai W, Zhou Y, Zhao J, Fang C, Yang R, Liu C. 2014. Phenotypic, genomic, transcriptomic and proteomic changes in Bacillus cereus after a short-term space flight. Adv Space Res 53:18–29. doi:10.1016/j.asr.2013.08.001.

[5] Wang Y, Yuan Y, Liu J, Su L, Chang D, Guo Y, Chen Z, Fang X, Wang J, Li T, Zhou L, Fang C, Yang R, Liu C. 2014. Transcriptomic and proteomic responses of Serratia marcescens to spaceflight conditions involve large-scale changes in metabolic pathways. Adv Space Res 53:1108–1117. doi:10.1016/j.asr.2014.01.018.

[6] Bai P, Zhang B, Zhao X, Li D, Yu Y, Zhang X, Huang B, Liu C. 2019. Decreased metabolism and increased tolerance to extreme environments in Staphylococcus warneri during long-term spaceflight. Microbiologyopen 8:e917. doi:10.1002/mbo3.917. 31414557 PMC6925155

[7] Qi JJ, Ma RC, Chen XD, Lan J. 2003. Analysis of genetic variation in Ganoderma lucidum after space flight. Adv Space Res 31:1617–1622. doi:10.1016/s0273-1177(03)00082-6. 12971418

[8] Wang Y, Wu J, Lv M, Shao Z, Hungwe M, Wang J, Bai X, Xie J, Wang Y, Geng W. 2021. Metabolism characteristics of lactic acid bacteria and the expanding applications in food industry. Front Bioeng Biotechnol 9:612285. doi:10.3389/fbioe.2021.612285. 34055755 PMC8149962

[9] Patel AK, Michaud P, Singhania RR, Soccol CR, Pandey A. 2010. Polysaccharides from probiotics: new developments as food additives. Food Technol Biotechnol 48:451–463.

[10] Oleksy M, Klewicka E. 2018. Exopolysaccharides produced by Lactobacillus sp.: biosynthesis and applications. Crit Rev Food Sci Nutr 58:450–462. doi:10.1080/10408398.2016.1187112. 27246190

[11] Tomcsik J, Baumann-Grace JB. 1959. Polysaccharide capsule of Bacillus megaterium. Proc Soc Exp Biol Med 101:570–571. doi:10.3181/00379727-101-25019. 13675320

[12] Hussain A, Zia KM, Tabasum S, Noreen A, Ali M, Iqbal R, Zuber M. 2017. Blends and composites of exopolysaccharides; properties and applications: a review. Int J Biol Macromol 94:10–27. doi:10.1016/j.ijbiomac.2016.09.104. 27697492

[13] Seesuriyachan P. 2012. Statistical modeling and optimization for exopolysaccharide production by Lactobacillus confusus in submerged fermentation under high salinity stress. Food Sci Biotechnol 21:1647–1654. doi:10.1007/s10068-012-0219-6. 22738958

[14] Yang F, Wang J, Zhang H, Xie Y, Jin J, Liu H, Pang X, Hao H. 2021. Hypoglycemic effects of space-induced Lactobacillus plantarum SS18-5 on type 2 diabetes in a rat model. J Food Biochem 45:e13899. doi:10.1111/jfbc.13899. 34396541

[15] Sun M-C, Hou P-P, Wang X-Y, Zhao C-H, Cheng B-J, Wang Y-L, Hao H-W, Zhang T-H, Ye H-Q. 2018. Pretreatment with Lactobacillus reuteri F-9–35 attenuates ethanol-induced gastric injury in rats. Food Nutr Res 62:1469. doi:10.29219/fnr.v62.1469. PMC6294840 30574053

[16] Sun M-C, Zhang F-C, Yin X, Cheng B-J, Zhao C-H, Wang Y-L, Zhang Z-Z, Hao H-W, Zhang T-H, Ye H-Q. 2018. Lactobacillus reuteri F-9-35 prevents DSS-induced colitis by inhibiting proinflammatory gene expression and restoring the gut microbiota in mice. J Food Sci 83:2645–2652. doi:10.1111/1750-3841.14326. 30216448

[17] Weng ML, Li JG, Gao F, Zhang XY, Wang PS, Jiang XC. 1999. The mutation induced by space conditions in Escherichia coli. World J Gastroenterol 5:445–447. doi:10.3748/wjg.v5.i5.445. 11819487 PMC4688619

[18] Hathi Z, Mettu S, Priya A, Athukoralalage S, Lam TN, Choudhury NR, Dutta NK, El-Omar EM, Gong L, Mohan G, Lin CSK. 2021. Methodological advances and challenges in probiotic bacteria production: ongoing strategies and future perspectives. Biochem Eng J 176:108199. doi:10.1016/j.bej.2021.108199.

[19] Liu W, Chen M, Duo L, Wang J, Guo S, Sun H, Menghe B, Zhang H. 2020. Characterization of potentially probiotic lactic acid bacteria and bifidobacteria isolated from human colostrum. J Dairy Sci 103:4013–4025. doi:10.3168/jds.2019-17602. 32113772

[20] Gao G, Ma T, Zhang T, Jin H, Li Y, Kwok L-Y, Zhang H, Sun Z. 2021. Adjunctive probiotic Lactobacillus rhamnosus Probio-M9 administration enhances the effect of anti-PD-1 antitumor therapy via restoring antibiotic-disrupted gut microbiota. Front Immunol 12:772532. doi:10.3389/fimmu.2021.772532. 34970262 PMC8712698

[21] Xu H, Hiraishi K, Kurahara L-H, Nakano-Narusawa Y, Li X, Hu Y, Matsuda Y, Zhang H, Hirano K. 2021. Inhibitory effects of breast milk-derived Lactobacillus rhamnosus Probio-M9 on colitis-associated carcinogenesis by restoration of the gut microbiota in a mouse model. Nutrients 13:1143. doi:10.3390/nu13041143. 33808480 PMC8065529

[22] Zheng Y, Yu Z, Zhang W, Sun T. 2021. Lactobacillus rhamnosus Probio-M9 improves the quality of life in stressed adults by gut microbiota. Foods 10:2384. doi:10.3390/foods10102384. 34681433 PMC8535744

[23] Mijakovic I, Poncet S, Boël G, Mazé A, Gillet S, Jamet E, Decottignies P, Grangeasse C, Doublet P, Le Maréchal P, Deutscher J. 2003. Transmembrane modulator-dependent bacterial tyrosine kinase activates UDP-glucose dehydrogenases. EMBO J 22:4709–4718. doi:10.1093/emboj/cdg458. 12970183 PMC212725

[24] Milojevic T, Weckwerth W. 2020. Molecular mechanisms of microbial survivability in outer space: a systems biology approach. Front Microbiol 11:923. doi:10.3389/fmicb.2020.00923. 32499769 PMC7242639

[25] Yu Y, Zhao X, Guo Y, Zhang X, Bai P, Zhang B, Wang J, Liu C. 2019. Identification of potential tobramycin-resistant mutagenesis of Escherichia coli strains after spaceflight. Future Microbiol 14:315–330. doi:10.2217/fmb-2018-0273. 30854896

[26] Zhao X, Yu Y, Zhang X, Huang B, Bai P, Xu C, Li D, Zhang B, Liu C. 2019. Decreased biofilm formation ability of Acinetobacter baumannii after spaceflight on China's Shenzhou 11 spacecraft. Microbiologyopen 8:e00763. doi:10.1002/mbo3.763. 30379419 PMC6562233

[27] Bryan RA, Zaragoza O, Zhang T, Ortiz G, Casadevall A, Dadachova E. 2005. Radiological studies reveal radial differences in the architecture of the polysaccharide capsule of Cryptococcus neoformans. Eukaryot Cell 4:465–475. doi:10.1128/EC.4.2.465-475.2005. 15701808 PMC549344

[28] Tynkkynen S, Buist G, Kunji E, Kok J, Poolman B, Venema G, Haandrikman A. 1993. Genetic and biochemical characterization of the oligopeptide transport system of Lactococcus lactis. J Bacteriol 175:7523–7532. doi:10.1128/jb.175.23.7523-7532.1993. 8244921 PMC206908

[29] Pinedo CA, Bringhurst RM, Gage DJ. 2008. Sinorhizobium meliloti mutants lacking phosphotransferase system enzyme HPr or EIIA are altered in diverse processes, including carbon metabolism, cobalt requirements, and succinoglycan production. J Bacteriol 190:2947–2956. doi:10.1128/JB.01917-07. 18281401 PMC2293241

[30] Prete R, Alam MK, Perpetuini G, Perla C, Pittia P, Corsetti A. 2021. Lactic acid bacteria exopolysaccharides producers: a sustainable tool for functional foods. Foods 10:1653. doi:10.3390/foods10071653. 34359523 PMC8305620

[31] Badel S, Bernardi T, Michaud P. 2011. New perspectives for lactobacilli exopolysaccharides. Biotechnol Adv 29:54–66. doi:10.1016/j.biotechadv.2010.08.011. 20807563

[32] Angelin J, Kavitha M. 2020. Exopolysaccharides from probiotic bacteria and their health potential. Int J Biol Macromol 162:853–865. doi:10.1016/j.ijbiomac.2020.06.190. 32585269 PMC7308007

[33] Sørensen HM, Rochfort KD, Maye S, MacLeod G, Brabazon D, Loscher C, Freeland B. 2022. Exopolysaccharides of lactic acid bacteria: production, purification and health benefits towards functional food. Nutrients 14:2938. doi:10.3390/nu14142938. 35889895 PMC9319976

[34] Lebeer S, Verhoeven TLA, Francius G, Schoofs G, Lambrichts I, Dufrêne Y, Vanderleyden J, De Keersmaecker SCJ. 2009. Identification of a gene cluster for the biosynthesis of a long, galactose-rich exopolysaccharide in Lactobacillus rhamnosus GG and functional analysis of the priming glycosyltransferase. Appl Environ Microbiol 75:3554–3563. doi:10.1128/AEM.02919-08. 19346339 PMC2687306

[35] Peant B, LaPointe G, Gilbert C, Atlan D, Ward P, Roy D. 2005. Comparative analysis of the exopolysaccharide biosynthesis gene clusters from four strains of Lactobacillus rhamnosus. Microbiology (Reading) 151:1839–1851. doi:10.1099/mic.0.27852-0. 15941992

[36] Islam ST, Lam JS. 2013. Wzx flippase-mediated membrane translocation of sugar polymer precursors in bacteria. Environ Microbiol 15:1001–1015. doi:10.1111/j.1462-2920.2012.02890.x. 23016929

[37] Ghosh P, Luong TT, Shah M, Thach TT, Choi S, Lee S, Rhee D-K. 2018. Adenylate kinase potentiates the capsular polysaccharide by modulating Cps2D in Streptococcus pneumoniae D39. Exp Mol Med 50:1–14. doi:10.1038/s12276-018-0141-y. PMC6123713 30185778

[38] Bender MH, Cartee RT, Yother J. 2003. Positive correlation between tyrosine phosphorylation of CpsD and capsular polysaccharide production in Streptococcus pneumoniae. J Bacteriol 185:6057–6066. doi:10.1128/JB.185.20.6057-6066.2003. 14526017 PMC225014

[39] Bender MH, Yother J. 2001. CpsB is a modulator of capsule-associated tyrosine kinase activity in Streptococcus pneumoniae. J Biol Chem 276:47966–47974. doi:10.1074/jbc.M105448200. 11606571

[40] Morona JK, Paton JC, Miller DC, Morona R. 2000. Tyrosine phosphorylation of CpsD negatively regulates capsular polysaccharide biosynthesis in Streptococcus pneumoniae. Mol Microbiol 35:1431–1442. doi:10.1046/j.1365-2958.2000.01808.x. 10760144

[41] Toniolo C, Balducci E, Romano MR, Proietti D, Ferlenghi I, Grandi G, Berti F, Ros IMY, Janulczyk R. 2015. Streptococcus agalactiae capsule polymer length and attachment is determined by the proteins CpsABCD. J Biol Chem 290:9521–9532. doi:10.1074/jbc.M114.631499. 25666613 PMC4392257

[42] Freitas F, Alves VD, Reis MAM. 2011. Advances in bacterial exopolysaccharides: from production to biotechnological applications. Trends Biotechnol 29:388–398. doi:10.1016/j.tibtech.2011.03.008. 21561675

[43] Kang H-J, Gilbert C, Badeaux F, Atlan D, LaPointe G. 2015. A tyrosine phosphorylation switch controls the interaction between the transmembrane modulator protein Wzd and the tyrosine kinase Wze of Lactobacillus rhamnosus. BMC Microbiol 15:40. doi:10.1186/s12866-015-0371-2. 25885688 PMC4340800

[44] Kalynych S, Valvano MA, Cygler M. 2012. Polysaccharide co-polymerases: the enigmatic conductors of the O-antigen assembly orchestra. Protein Eng Des Sel 25:797–802. doi:10.1093/protein/gzs075. 23100544

[45] Islam ST, Huszczynski SM, Nugent T, Gold AC, Lam JS. 2013. Conserved-residue mutations in Wzy affect O-antigen polymerization and Wzz-mediated chain-length regulation in Pseudomonas aeruginosa PAO1. Sci Rep 3:3441. doi:10.1038/srep03441. 24309320 PMC3854497

[46] Berghoff BA, Konzer A, Mank NN, Looso M, Rische T, Förstner KU, Krüger M, Klug G. 2013. Integrative “omics”-approach discovers dynamic and regulatory features of bacterial stress responses. PLoS Genet 9:e1003576. doi:10.1371/journal.pgen.1003576. 23818867 PMC3688512

[47] Bathke J, Konzer A, Remes B, McIntosh M, Klug G. 2019. Comparative analyses of the variation of the transcriptome and proteome of Rhodobacter sphaeroides throughout growth. BMC Genomics 20:358. doi:10.1186/s12864-019-5749-3. 31072330 PMC6509803

[48] Taniguchi Y, Choi PJ, Li G-W, Chen H, Babu M, Hearn J, Emili A, Xie XS. 2010. Quantifying E-coli proteome and transcriptome with single-molecule sensitivity in single cells. Science 329:533–538. doi:10.1126/science.1188308. 20671182 PMC2922915

[49] Goh KKT, Haisman RD, Singh H. 2006. Examination of exopolysaccharide produced by Lactobacillus delbrueckii subsp bulgaricus using confocal laser scanning and scanning electron microscopy techniques. J Food Sci 70:M224–M229. doi:10.1111/j.1365-2621.2005.tb07192.x.

[50] Breakwell DP, Moyes RB, Reynolds J. 2009. Differential staining of bacteria: capsule stain. Curr Protoc Microbiol 15:A.3I.1–A.3I.4. doi:10.1002/9780471729259.mca03is15. 19885936

[51] Lim HJ, Lee EH, Yoon Y, Chua B, Son A. 2016. Portable lysis apparatus for rapid single-step DNA extraction of Bacillus subtilis. J Appl Microbiol 120:379–387. doi:10.1111/jam.13011. 26606545

[52] Luo R, Liu B, Xie Y, Li Z, Huang W, Yuan J, He G, Chen Y, Pan Q, Liu Y, Tang J, Wu G, Zhang H, Shi Y, Liu Y, Yu C, Wang B, Lu Y, Han C, Cheung DW, Yiu S-M, Peng S, Xiaoqian Z, Liu G, Liao X, Li Y, Yang H, Wang J, Lam T-W, Wang J. 2012. SOAPdenovo2: an empirically improved memory-efficient short-read de novo assembler. Gigascience 1:18. doi:10.1186/2047-217X-1-18. 23587118 PMC3626529

[53] Hunt M, Silva ND, Otto TD, Parkhill J, Keane JA, Harris SR. 2015. Circlator: automated circularization of genome assemblies using long sequencing reads. Genome Biol 16:294. doi:10.1186/s13059-015-0849-0. 26714481 PMC4699355

[54] Walker BJ, Abeel T, Shea T, Priest M, Abouelliel A, Sakthikumar S, Cuomo CA, Zeng Q, Wortman J, Young SK, Earl AM. 2014. Pilon: an integrated tool for comprehensive microbial variant detection and genome assembly improvement. PLoS One 9:e112963. doi:10.1371/journal.pone.0112963. 25409509 PMC4237348

[55] Kurtz S, Phillippy A, Delcher AL, Smoot M, Shumway M, Antonescu C, Salzberg SL. 2004. Versatile and open software for comparing large genomes. Genome Biol 5:R12–R19. doi:10.1186/gb-2004-5-2-r12. 14759262 PMC395750

[56] Alikhan NF, Petty NK, Ben Zakour NL, Beatson SA. 2011. BLAST Ring Image Generator (BRIG): simple prokaryote genome comparisons. BMC Genomics 12:402. doi:10.1186/1471-2164-12-402. 21824423 PMC3163573

[57] Seemann T. 2014. Prokka: rapid prokaryotic genome annotation. Bioinformatics 30:2068–2069. doi:10.1093/bioinformatics/btu153. 24642063

[58] Reeves PR, Hobbs M, Valvano MA, Skurnik M, Whitfield C, Coplin D, Kido N, Klena J, Maskell D, Raetz CR, Rick PD. 1996. Bacterial polysaccharide synthesis and gene nomenclature. Trends Microbiol 4:495–503. doi:10.1016/s0966-842x(97)82912-5. 9004408

[59] Price MN, Dehal PS, Arkin AP. 2009. FastTree: computing large minimum evolution trees with profiles instead of a distance matrix. Mol Biol Evol 26:1641–1650. doi:10.1093/molbev/msp077. 19377059 PMC2693737

[60] Dubois M, Gilles KA, Hamilton JK, Rebers PA, Smith F. 1956. Colorimetric method for determination of sugars and related substances. Anal Chem 28:350–356. doi:10.1021/ac60111a017.

[61] Langmead B, Salzberg SL. 2012. Fast gapped-read alignment with Bowtie 2. Nat Methods 9:357–359. doi:10.1038/nmeth.1923. 22388286 PMC3322381

[62] Love M, Anders S, Huber W. 2014. Differential analysis of count data–the DESeq2 package. Genome Biol 15:550. doi:10.1186/s13059-014-0550-8. 25516281 PMC4302049

[63] E JJ, Ma LL, Chen ZC, Ma RZ, Zhang QL, Sun RY, et al. 2020. Effects of buffer salts on the freeze-drying survival rate of Lactobacillus plantarum LIP-1 based on transcriptome and proteome analyses. Food Chem 326:126849. doi:10.1016/j.foodchem.2020.126849. 32447159

[64] Cao C, Wang J, Liu Y, Kwok L-Y, Zhang H, Zhang W. 2020. Adaptation of Lactobacillus plantarum to ampicillin involves mechanisms that maintain protein homeostasis. mSystems 5:e00853-19. doi:10.1128/mSystems.00853-19. 31992633 PMC6989132

[65] Yu GC, Smith DK, Zhu HC, Guan Y, Lam TTY. 2017. GGTREE: an R package for visualization and annotation of phylogenetic trees with their covariates and other associated data. Methods Ecol Evol 8:28–36. doi:10.1111/2041-210X.12628.

