# Supplementary material for: The Space Environment Activates Capsular Polysaccharide Production in Lacticaseibacillus rhamnosus Probio-M9 by Mutating the wze (ywqD) Gene
Source: Microbiol Spectr. 2023 Mar 2;11(2):e04677-22. doi: 10.1128/spectrum.04677-22 (PMC10101077; doi:10.1128/spectrum.04677-22)

Fig. S1 Growth of space mutants on de Man, Rogosa and Sharpe agar for 48 hours. The colonies showed different colony size and morphology.

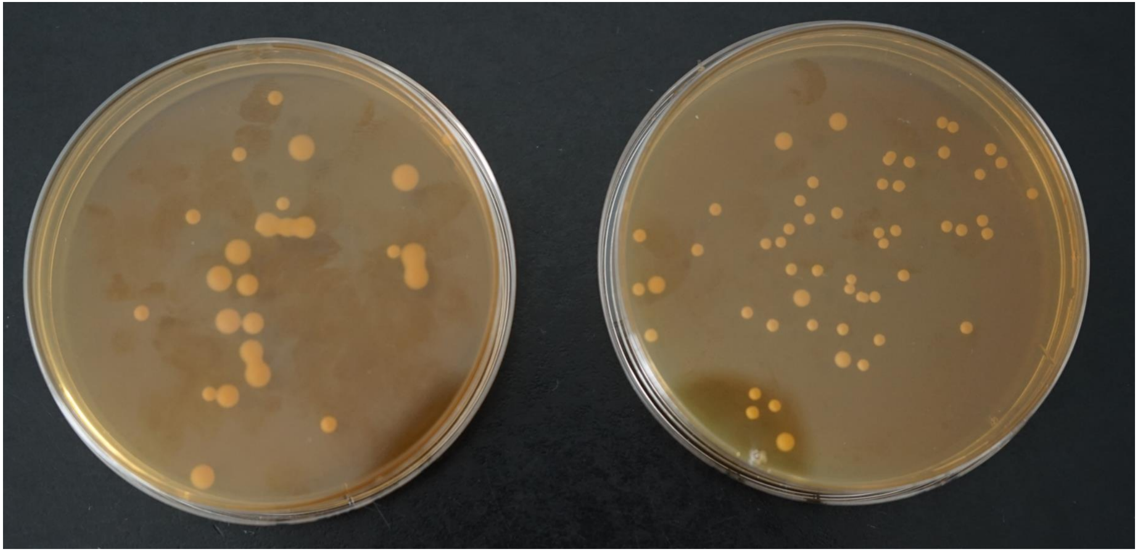

Space mutant (glycerol) Space mutant (MRS agar)

Fig. S2 Venn diagrams showing the number of common or unique genes (a) and proteins (b) of significant differential expression (up = up-regulation; down = down-regulation) in the space mutants (HG-R7970-3 and HG-R7970-41) in comparison with a ground control isolate (SG-R7970-16).

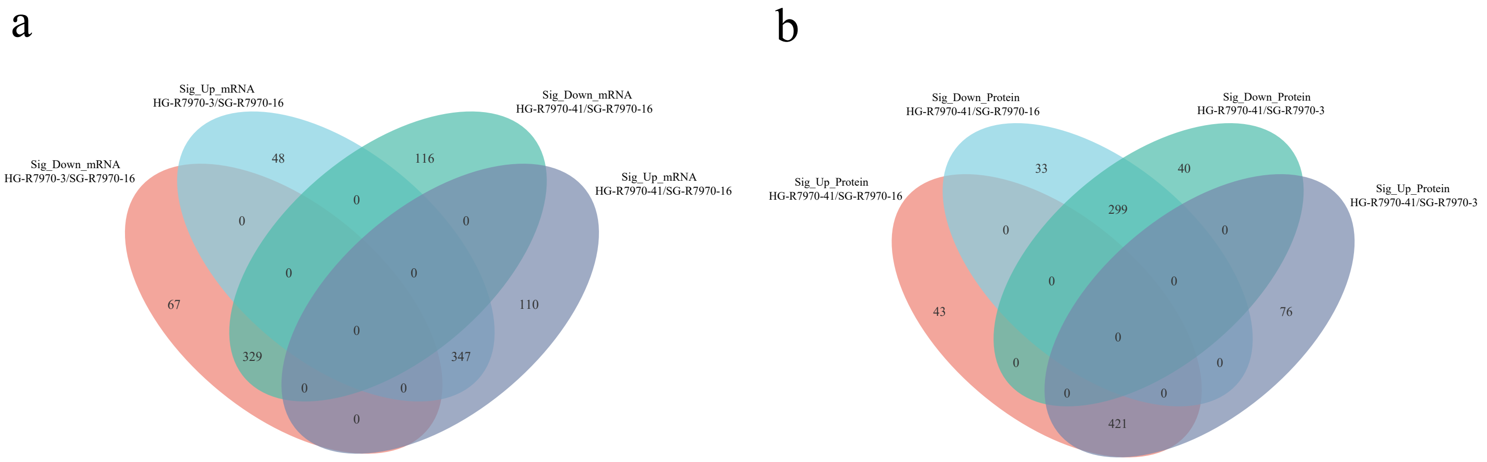

Supplement: Supplemental file 3 — Supplemental material. Download spectrum.04677-22-s0003.pdf, PDF file, 0.4 MB [file spectrum.04677-22-s0003.pdf]
